# Cross-linking and modification of fibronectin by peroxynitrous acid: Mapping and quantification of damage provides a new model for domain interactions

**DOI:** 10.1016/j.jbc.2021.100360

**Published:** 2021-02-02

**Authors:** Michele Mariotti, Adelina Rogowska-Wrzesinska, Per Hägglund, Michael J. Davies

**Affiliations:** 1 Department of Biomedical Sciences, Panum Institute, University of Copenhagen, Copenhagen, Denmark; 2 Department of Biochemistry and Molecular Biology and VILLUM Center for Bioanalytical Sciences, University of Southern Denmark, Odense, Denmark

**Keywords:** fibronectin, extracellular matrix, cross-links, nitration, oxidation, peroxynitrous acid, di-tyrosine, 3-nitrotyrosine, 6-nitrotryptophan, 3-nitroTyr, 3-nitrotyrosine, di-Tyr, *o,o*-di-tyrosine, DTT, dithiothreitol, ECM, extracellular matrix, FN, fibronectin, HOCl, the physiological mixture of hypochlorous acid and its conjugate anion ^−^OCl, MPO, myeloperoxidase, ONOOH, the physiological mixture of peroxynitrous acid and its conjugate anion ONOO^−^, RSO, relative site occupancy, SEC, size exclusion chromatography

## Abstract

Fibronectin (FN) is an abundant glycoprotein found in plasma and the extracellular matrix (ECM). It is present at high concentrations at sites of tissue damage, where it is exposed to oxidants generated by activated leukocytes, including peroxynitrous acid (ONOOH) formed from nitric oxide (from inducible nitric oxide synthase) and superoxide radicals (from NADPH oxidases and other sources). ONOOH reacts rapidly with the abundant tyrosine and tryptophan residues in ECM proteins, resulting in the formation of 3-nitrotyrosine, di-tyrosine, and 6-nitrotryptophan. We have shown previously that human plasma FN is readily modified by ONOOH, but the *extent* and *location* of modifications, and the role of FN structure (compact *versus* extended) in determining these factors is poorly understood. Here, we provide a detailed LC-MS analysis of ONOOH-induced FN modifications, including the extent of their formation and the sites of intramolecular and intermolecular cross-links, including Tyr-Tyr, Trp-Trp, and Tyr-Trp linkages. The localization of these cross-links to specific domains provides novel data on the interactions between different modules in the compact conformation of plasma FN and allows us to propose a model of its unknown quaternary structure. Interestingly, the pattern of modifications is significantly different to that generated by another inflammatory oxidant, HOCl, in both extent and sites. The characterization and quantification of these modifications offers the possibility of the use of these materials as specific biomarkers of ECM modification and turnover in the many pathologies associated with inflammation-associated fibrosis.

Fibronectin (FN) is an abundant glycoprotein present both in plasma and the extracellular matrix (ECM) (1, 2). It is composed of two very similar subunits, each of 230 to 270 kDa that are linked to form a dimeric structure by two asymmetric disulfide bonds located near the carboxyl termini (1, 2). Each monomer is composed of a series of type I, II, and III modules that are organized into multimodular structural domains with affinity for macromolecules such as fibrin, heparin, collagen, and integrin (Fig. 1). The plasma form is synthesized by hepatocytes and subsequently exported to the plasma where it circulates as a dimer in a compact conformation stabilized through electrostatic interactions between different modules (3). This structural arrangement is thought to prevent unwanted interactions with other proteins (3, 4) and may also serve to protect FN from damage caused by inflammatory oxidants. Conversion from the compact conformation to an extended form of FN as well as partial unfolding of the protein occur when the plasma form is incorporated into blood clots, and when the cell-derived form generates fibrils in the ECM, with the latter process believed to be initiated, at least in part by mechanotransductive forces induced by cells (5, 6, 7). Conversion of the compact conformation to the extended form of FN can also be induced *in vitro* by high salt concentrations, or changes in pH, which disrupt electrostatic interactions (8, 9). Plasma FN plays a key role in the early physiological responses at sites of tissue injury as it is a key component of blood clots. This occurs *via* binding to fibrin fibers, *via* multiple binding domains, to give a temporary matrix that promotes hemostasis. This matrix supports the migration and adhesion of fibroblast and endothelial cells which replace the provisional matrix over time with collagens, laminins, cell-derived FN, and other ECM components (4, 10, 11). The form present in tissue ECM is synthesized by endothelial, smooth muscle, and fibroblast cells, amongst others, and is structurally related to the plasma isoform but contains extra domains (extra domains A and B) that are alternatively spliced type III modules (4, 10, 11, 12); these are not present in the plasma form. Furthermore, in contrast to the soluble plasma form, the ECM FN is polymerized into elastic fibrils which are attached to the cell membrane through integrins and provides binding sites for other ECM components such as collagen, heparin, and proteoglycans. The detailed mechanism of fibril formation is not established, but it is thought to involve conformational remodeling of FN dimers from the compact to extended conformations and partial unfolding of type III modules through mechanical forces mediated by the actin cytoskeleton (6, 7).

**Figure 1:**
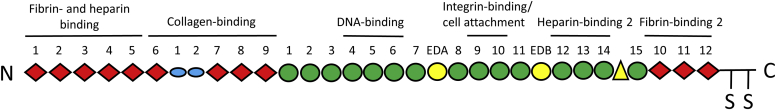
**Schematic overview of the multimodular structure of a FN monomer with the positions of the functional domains and the two intermolecular disulfide bonds indicated.** Type I, II, and III modules are numbered from the N termini to the C termini and displayed as *red diamonds*, *blue ovals*, and *green circles*, respectively. The alternatively spliced type III modules (EDA/EDB; only present in cell-derived FN) and the variable region are shown as *yellow circles* and *triangle*, respectively. FN, fibronectin.

FN is present in the ECM of many tissues and particularly in basement membranes, under normal physiological conditions (13), but a significant increase in FN concentration and changes in the isoforms occur in response to tissue injury. Thus, higher levels have been reported in heart failure (14), in ischemic heart disease (15), and in atherosclerotic lesions of the artery wall during the development of cardiovascular disease (16). In heart failure, inhibition of FN accumulation has been reported to attenuate fibrosis and improve cardiac function (14), and a deficiency of plasma FN impedes the development of atherosclerotic lesions and fibrous cap formation (17, 18). The presence of FN in atherosclerotic lesions may however be a “double-edged” sword (19), as the presence of this material promotes the formation of a thick and stable fibrous cap, which is associated with a lower risk of lesion rupture and consequent heart attack or stroke (20). These data are consistent with the hypothesis that FN plays a critical role in both promoting the initial accumulation of lipids and (proinflammatory) macrophages within the artery wall and subsequently the generation and stabilization of the fibrous cap (17). The colocalization of inflammatory cells with FN in the shoulder regions of plaques, and FN degradation during the progression of atherosclerosis, support the hypothesis that lesion integrity and stability is affected by species, both oxidants and enzymes, released by inflammatory cells, with this occurring at least in part *via* ECM damage (21, 22).

Macrophages and leukocytes generate oxidants intentionally during the innate immune response to invading pathogens (23). While neutrophils and monocytes generate high yields of the potent bactericide hypochlorous acid (HOCl) *via* the heme enzyme myeloperoxidase (MPO), most macrophages express little or none of this enzyme. In contrast, on exposure to cytokines, macrophages upregulate the synthesis of the inducible nitric oxide synthase isoform (iNOS, NOS2) that forms relatively high levels of nitric oxide (NO^**.**^). Reaction of this species with superoxide radicals (O_2_^**−.**^) formed by NADPH oxidase enzymes, as well as other sources, gives rise to the potent oxidant and nitrating species, peroxynitrous acid and its conjugate anion (ONOOH) (24, 25). Although both ONOOH and HOCl play an important role in protection against invading pathogens, unintended (sterile inflammation) or excessive formation of these species can result in host cell and tissue damage. Such changes have been associated with many of the human pathologies that have an acute or chronic inflammatory component, including atherosclerosis (26, 27, 28, 29, 30).

As these oxidants are released extracellularly and the ECM is poorly protected against modification because of much lower levels of protective antioxidants, enzymes and repair systems when compared with cells (31), it might be expected that ECM components would be both major targets, and accumulate damage because of their relatively long half-lives (21, 32). This would also be expected to be compounded by the high levels of ECM materials in many tissues and particularly in major arteries. This hypothesis is supported by studies that have shown co-localization of ECM species with biomarkers of ONOOH-mediated damage (22, 33, 34, 35). Such damage may have functional implications, as cells adhere to ONOOH-modified FN to a much lower extent than native FN, with this occurring in a dose-dependent manner with increasing ONOOH exposure (33).

ONOOH can either induce oxidation directly or undergo reactions to form other reactive species (25, 36). Thus, ONOOH can decompose (to a limited extent) to the reactive radicals HO^**.**^ and NO_2_^**.**^ or react with CO_2_ to form peroxynitrosocarbonate (ONOOCO_2_^−^); the latter can, in turn, decompose to NO_2_^**.**^ and CO_3_^−**.**^ (25). ONOOH reacts at significant rates with cysteine and methionine (25, 36), but many ECM proteins contain relatively modest levels of these species, and cysteine in particular; reaction with the disulfide species cystine is less rapid (36). ONOOH also reacts at considerable rates with tyrosine (Tyr) and tryptophan (Trp) residues (36), and ECM proteins often contain significant levels of these residues, resulting in these being major targets for ONOOH and ONOOCO_2_^−^. These reactions result in the formation of 3-nitroTyr and the cross-linked species di-tyrosine (di-Tyr) from Tyr, and 6-nitrotryptophan (6-nitroTrp) from Trp (25, 36). In a previous study, we have shown that human plasma FN is readily modified by ONOOH (and ONOOCO_2_^−^) with this resulting in significant levels of these modified amino acids, as well as cross-linked materials. However, although the *extent* of modification was quantified, the *locations* of these alterations within the protein sequence were not determined (33). In the study reported here, we provide a detailed analysis of the location of modifications formed on Met, Tyr, and Trp (as major targets for ONOOH) within the FN structure and their relative extents of alteration, as well as identifying the sites of intramolecular and intermolecular cross-links. The localization of these cross-links also provides novel data on the interactions between different modules in the compact conformation of plasma FN which allows us to propose schematic models of its quaternary structure. The pattern of these modifications is also compared with those observed with another inflammatory oxidant, HOCl (37), and shown to be markedly different.

## Results

### An experimental model to study the impact of conformational flexibility on the oxidative footprint of FN exposed to ONOOH

There is strong literature evidence based on microscopy and biophysical methods suggesting that FN circulates in plasma in a compact conformation that is stabilized by electrostatic interaction between modules (8, 9). By modulating pH or salt concentrations, it is possible to break these electrostatic interactions and reversibly switch FN to an extended conformation with a larger hydrodynamic radius. This major structural rearrangement can be expected to modulate the susceptibility of the protein to oxidants such as peroxynitrous acid, which is known to induce nitration and cross-linking in FN and other ECM proteins. We hypothesized that a (simple) experimental model where nitration sites and cross-links in FN exposed to ONOOH in a low (150 mM) and high (750 mM) NaCl concentration are identified by mass spectrometry may provide an “oxidative footprint” which can reveal important information about the impact of the tertiary structure on the susceptibility of FN to ONOOH. In the extended conformation, a greater accessibility of oxidants to regions of the FN structure that are buried in the compact conformation might be expected. Gel filtration chromatography (SEC) of native FN incubated in phosphate buffer supplemented with 150 mM NaCl and 750 mM, respectively, demonstrates a shorter retention time for the latter, in agreement with a larger hydrodynamic radius of the extended conformation (Fig. S1).

To demonstrate the feasibility of the approach, LC-MS analysis of control and ONOOH-treated protein samples (final protein concentration 1 μM) was performed in buffer containing 150 mM NaCl, after tryptic digestion, which allowed the identification of peptides from most of the human FN isoform 1 sequence (Uniprot ID P02751), with coverage being 74.1% for the control protein (80.7% when glycosylated residues are excluded). Similar or slightly higher levels of coverage were detected for the oxidant treated protein (Table 1). The majority of the Tyr and Trp in the FN sequence were detected, with 76 of the 100 Tyr residues (76%) and 33 of 39 Trp residues (84.6%) detected for the control protein (Table 1). For the protein modified by 50 μM or 500 μM ONOOH, the number of Trp residues detected remained constant, whereas the numbers for Tyr increased with 83% detected (Table 1). This increase is probably because of oxidation-induced unfolding of the protein structure allowing greater access to the trypsin used for digestion. Similar coverage data were obtained for samples run in buffer containing 750 mM NaCl (data not shown).

**Table 1:** Sequence coverage (with and without the inclusion of glycosylation sites) of control and ONOOH (50 μM or 500 μM) treated FN (1 μM)

Coverage of protein, and Tyr (Y) and Trp (W) residues	Control	50 μM ONOOH		500 μM ONOOH
		Normal salt (150 mM NaCl)	High salt (750 mM NaCl)	
Sequence coverage	74.1%	74.1%	74.1%	76.2%
Sequence coverage excluding known glycosylation sites	80.7%	80.7%	80.7%	82.8%
Y total	100	100	100	100
Y nitrated	0	46 (46%)	47 (47%)	46 (46%)
Y not nitrated	76 (76%)	37 (37%)	36 (36%)	37 (37%)
Y not identified	24 (24%)	17 (17%)	17 (17%)	17 (17%)
W total	39	39	39	39
W nitrated	0	8 (20.5%)	9 (23.1%)	8 (20.5%)
W not nitrated	33 (84.6%)	25 (64.1%)	24 (61.5%)	25 (64.1%)
W not identified	6 (15.4%)	6 (15.4%)	6 (15.4%)	6 (15.4%)

FN, fibronectin; ONOOH, peroxynitrous acid and its conjugate anion.

The number of total tyrosine (Tyr, Y) and tryptophan (Trp, W), nitrated, not nitrated and not identified Tyr and Trp residues are indicated, including the percentage of these residues (data in parentheses).

The number and position of nitration modifications in the protein sequence and the abundance of these modifications within the detected sequence were subsequently analyzed. After treatment with 50 μM ONOOH in buffer containing 150 mM NaCl, 46% of the total Tyr residues (54% of those identified) were detected as nitrated, with 37% detected as not nitrated and 17% not identified (Table 1). Similar levels were detected for the 500 μM condition. Corresponding treatment of FN with 50 μM ONOOH in buffer supplemented with 750 mM NaCl yielded two additional nitration sites (one Tyr and one Trp) when compared with the 150 mM NaCl condition. For Trp residues, the number and percentage of nitrated Trp residues was significantly lower than for Tyr, corresponding to 20.5% of the total (24.2% of those identified), with 64.1% of the total Trp residues identified being not nitrated, and 15.4% of the Trp residues not detected in either form for the 50 μM ONOOH condition. No Tyr and Trp nitration site was detected in the control (nonoxidized) samples (Table 1).

### Mapping of amino acid nitration sites

The extent of modification at each of the Tyr and Trp residues identified as sites of modification was quantified by determining the % modification at each site relative to all peptides that contained this site (*i.e.*, relative site occupancy, RSO), including those with Met oxidation. Data were determined for FN subjected to oxidation by ONOOH (either 50 or 500 μM), with the reactions carried out either in the presence of normal (physiological) NaCl concentrations (150 mM) or under high salt concentrations (750 mM). The oxidized samples were subsequently reduced to the monomer chains by treatment with dithiothreitol (DTT) in the presence of SDS and separated by SEC in the presence of 0.1% SDS. The monomer chains, collected as a single fraction, were then subjected to LC-MS analysis.

**Figure 2:**
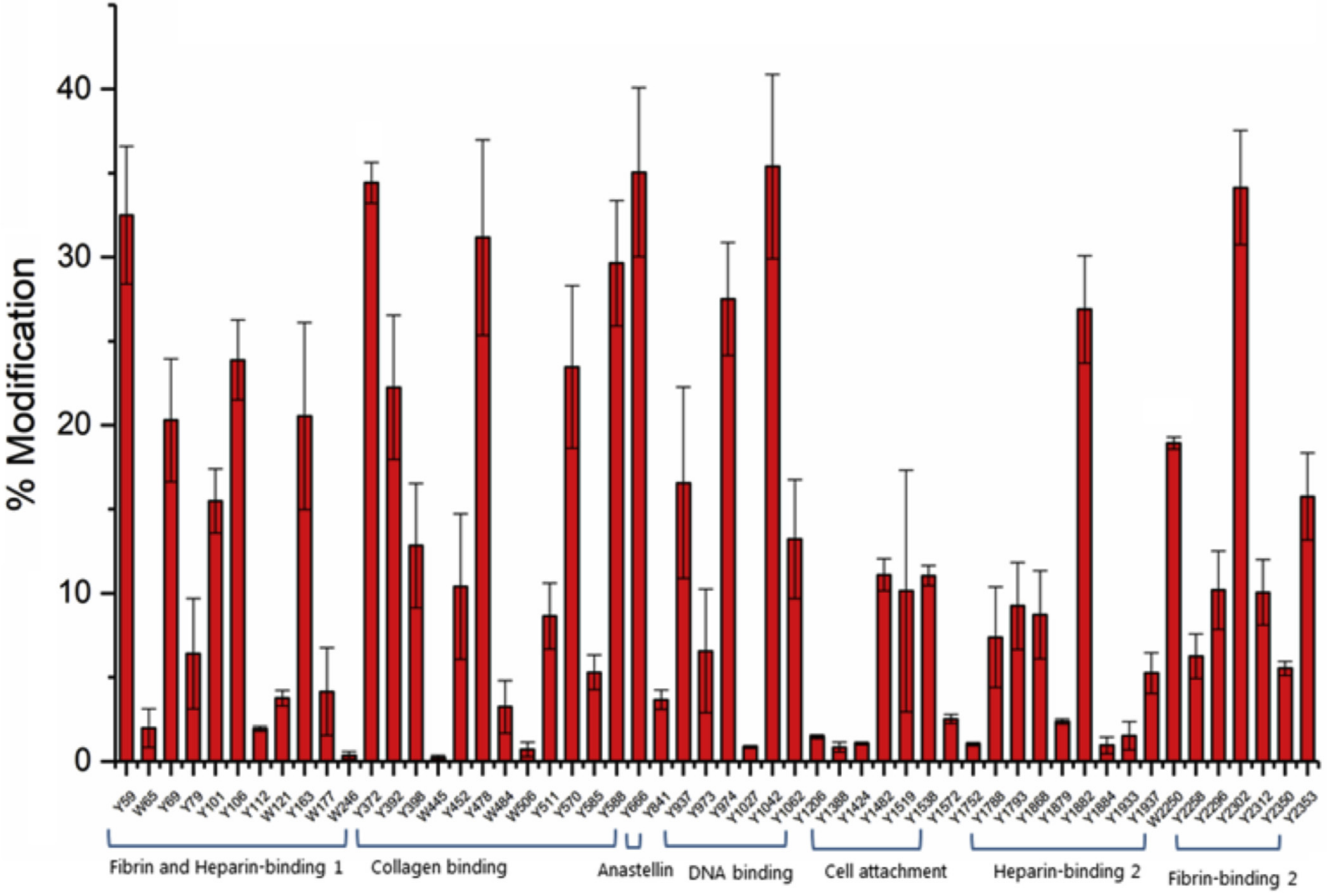
**Quantification of nitrated Tyr (Y) and Trp (W) in the monomeric fraction of FN (1 μM) after exposure to 50 μM ONOOH in buffer containing 150 mM NaCl and subsequent separation using gel filtration chromatography (SEC) under denaturing conditions with 0.1% SDS**. FN, fibronectin, ONOOH, peroxynitrous acid and its conjugate anion. Data are presented as mean ± SD from three independent experiments.

For FN treated with 50 μM ONOOH in the presence of 150 mM NaCl, a large variation in the extent of nitration was observed across the Tyr residues in the FN sequence with this ranging from 0 to ∼35% (Fig. 2). A smaller range of nitration extents was observed for the Trp residues with this ranging from 0 to ∼18%, though this distribution is skewed by the high RSO level of Trp2250 (W2250), with the majority of the Trp residues showing modification levels of <5%. In contrast, 14 Tyr residues showed heavy modification (RSO between 20 and 35%). The extent of nitration was particularly marked for Tyr59, Tyr372, Tyr452, Tyr666, Tyr1042, and Tyr2302, with each having a nitration level >30% (Fig. 2).

**Figure 3:**
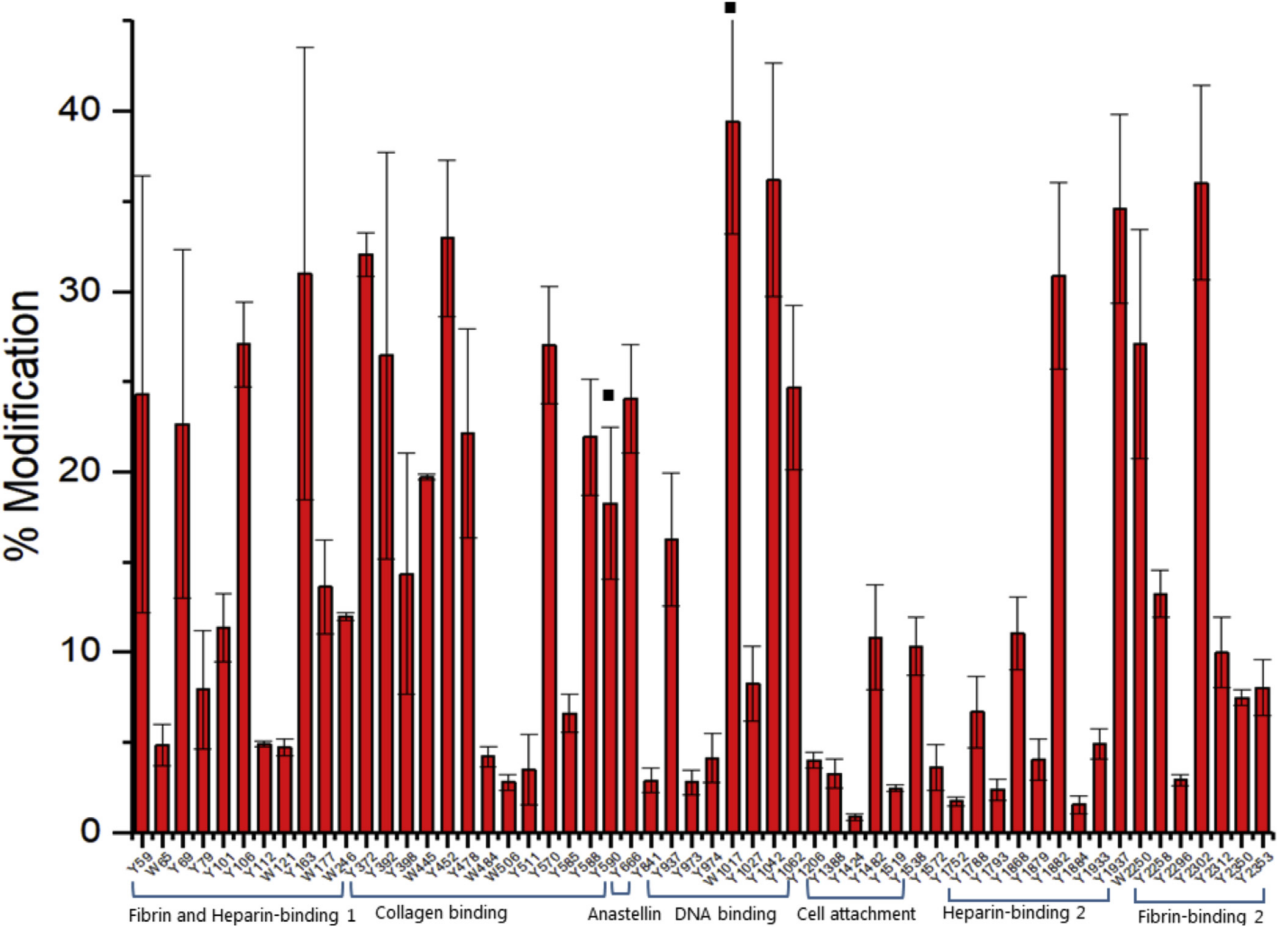
**Quantification of nitrated Tyr (Y) and Trp (W) in monomeric fraction of FN (1 μM) after exposure to 50 μM ONOOH in buffer containg 750 mM NaCl and separation using gel filtration chromatography (SEC) using denaturing conditions with 0.1% SDS**. ■ indicates the residues which are not found in FN exposed to 50 μM ONOOH in buffer containing 150 mM NaCl. FN, fibronectin; ONOOH, peroxynitrous acid and its conjugate anion. Data are presented as mean ± SD from three independent experiments.

**Figure 4:**
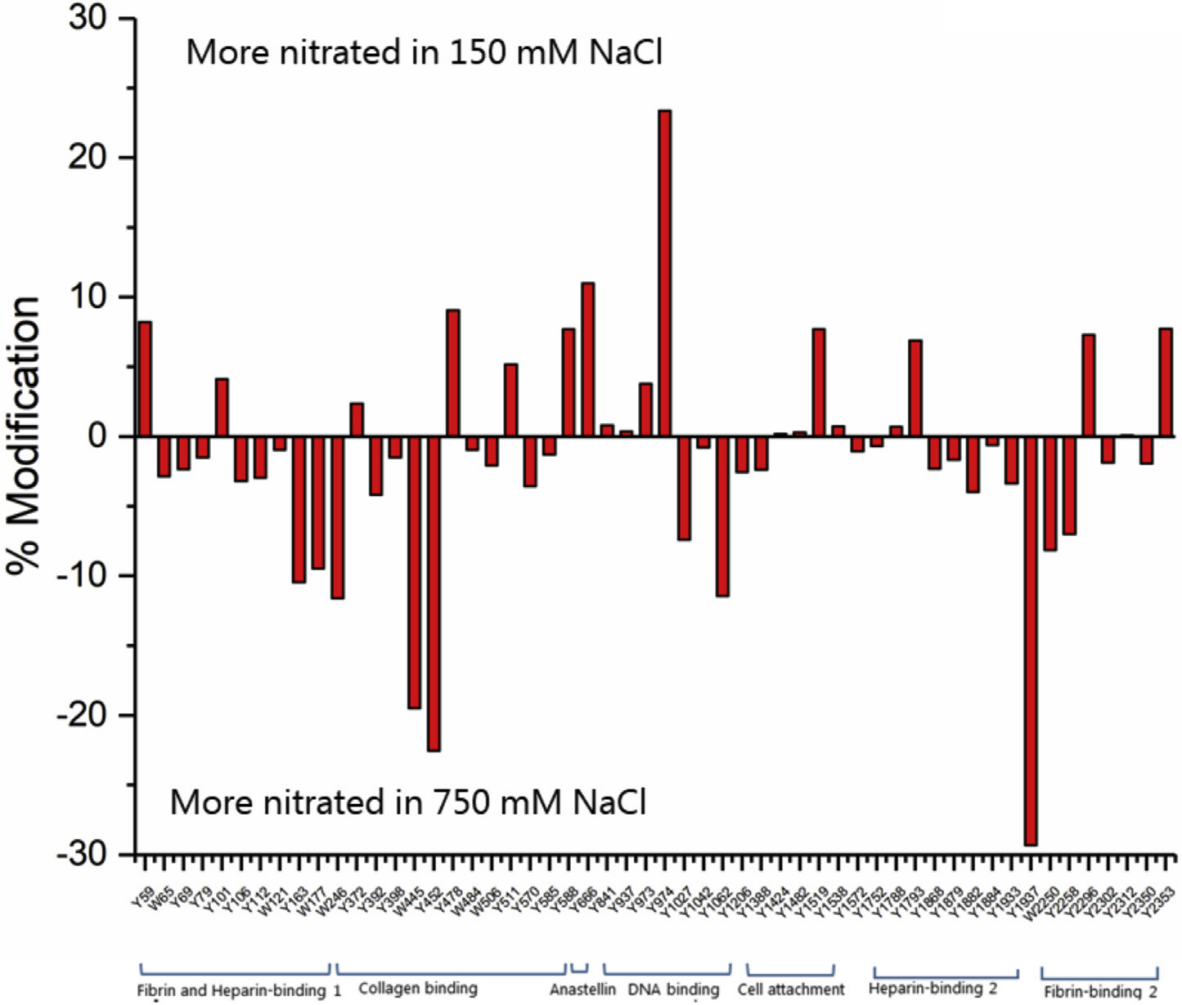
**Nitration levels at Tyr (Y) and Trp (W) for monomeric FN (1 μM) treated with 50 μM ONOOH in buffer containing 150 *versus* 750 mM NaCl, and then separated using gel filtration chromatography (SEC) in denaturing conditions with 0.1% SDS (see**Experimental procedures**).** Positive values indicate a greater extent of nitration at the residue under consideration for FN treated with ONOOH in buffer with 150 mM NaCl, whereas negative values indicate a greater extent of nitration for FN treated with ONOOH in buffer with 750 mM NaCl. For Tyr 590 and Trp 1017, a 0% modification level was assumed in the 150 mM NaCl dataset. FN, fibronectin; ONOOH, peroxynitrous acid and its conjugate anion.

For FN treated with 50 μM ONOOH in the presence of 750 mM NaCl (*i.e.*, with an extended FN conformation), the overall extent of nitration was slightly higher, increasing from a mean level of nitration (across the nitrated residues) of 12.1% for the samples containing 150 mM NaCl to a mean level of nitration of 13.6% for the samples containing 750 mM NaCl (Fig. 3). Figure 4 illustrates whether particular Tyr and Trp residues were more heavily nitrated in the low or high NaCl concentrations. These data indicate that there is more extensive nitration at a significant number of Tyr and Trp residues under the high salt (extended conformation) than the normal salt condition (*i.e.*, a greater number and extent below the mid-line in Fig. 4) but also that some residues were less extensively nitrated in the extended conformation (*i.e.*, the bars above the midline, Fig. 4). Thus, not all residues appear to be more exposed to the oxidant in the extended structure. Furthermore, these changes are not evenly distributed across the sequence, with the greatest enhancement in nitration under the high salt concentration observed for specific Tyr and Trp residues present in regions within the fibrin- and heparin-binding 1 domain, the collagen-binding domain, the DNA-binding domain, the heparin-binding 2 domain, and the fibrin-binding 2 domain, suggesting that these domains are more markedly affected by the high salt concentration. In particular, two residues—Tyr590 and Trp1017—that were identified, but not detected as nitrated in the 150 mM NaCl condition, were detected with extensive levels of modification (∼18% and ∼39% modification respectively), with the Trp1017 residue being the most heavily modified of all the residues detected in the high salt condition. In available crystal structures, Tyr590 from the FNI_9_ module of the collagen-binding domain is exposed on the surface, whereas Trp1017 is buried in the hydrophobic core of FNIII_5_ of the DNA-binding domain (see Discussion). Other modification sites in the DNA-binding domain showed mixed effects, with some residues being higher and others lower. In contrast, Tyr666 from the FNIII_1_ module (anastellin) showed a higher level of nitration when treated with ONOOH under the low (physiological) salt concentrations. Other regions, such as the cell-binding domain showed limited changes.

**Figure 5:**
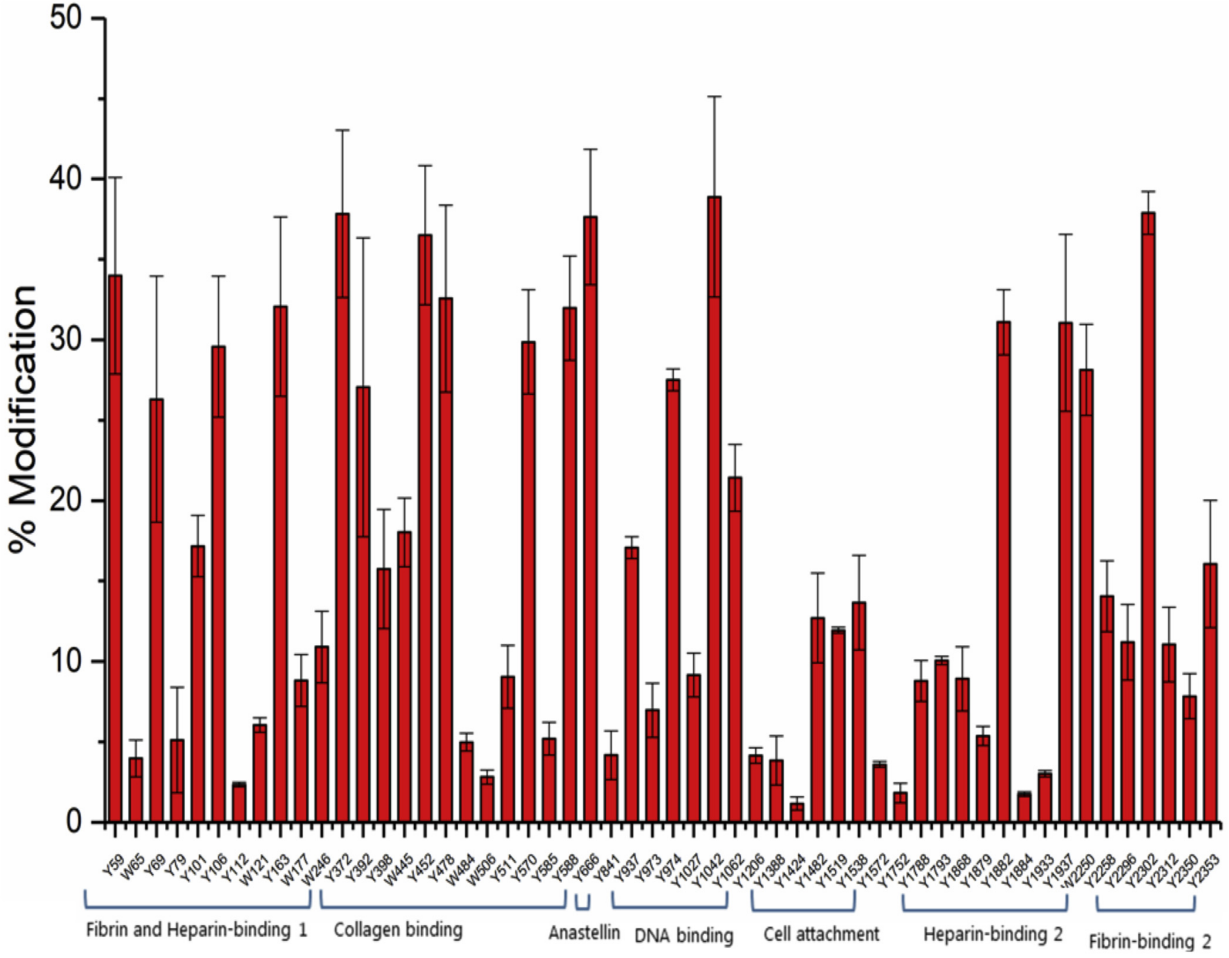
**Quantification of nitrated Tyr (Y) and Trp (W) in the monomer fraction of FN (1 μM) treated with 500 μM ONOOH in buffer containing 150 mM NaCl and subsequently separated using gel filtration chromatography (SEC) under denaturing conditions with 0.1% SDS**. FN, fibronectin; ONOOH, peroxynitrous acid and its conjugate anion. Data are presented as mean ± SD from three independent experiments.

When FN was treated with higher levels of ONOOH (500 μM), the overall and average level of nitration increased as might be expected from the higher oxidant dose, but many of the residues that were heavily nitrated at the lower concentration of oxidant did not show proportionately greater modification (*i.e.*, the level was not increased ten-fold, with the ten-fold increase in ONOOH concentration) (Fig. 5). However, many of the less heavily modified residues at the low ONOOH concentration, both Tyr and Trp, were modified to a much greater extent at the higher oxidant level, resulting in a much lower extent of variation and a more even distribution of damage (Fig. 5). Interestingly, the overall numbers of residues that were modified (*i.e.*, the number that were detected with any extent of modification) was not significantly increased with the high oxidant dose, suggesting that the lack of modification is not due merely to a limited supply of the oxidant but because of other factors such as steric shielding or other effects (*e.g.*, electronic interactions). In the presence of 750 mM NaCl, one more Trp residue was detected as nitrated in treatment with 50 μM ONOOH (Trp1017), which was not identified as nitrated with 500 μM (with 150 mM NaCl), though the nonmodified residue was detected under the latter conditions.

**Figure 6:**
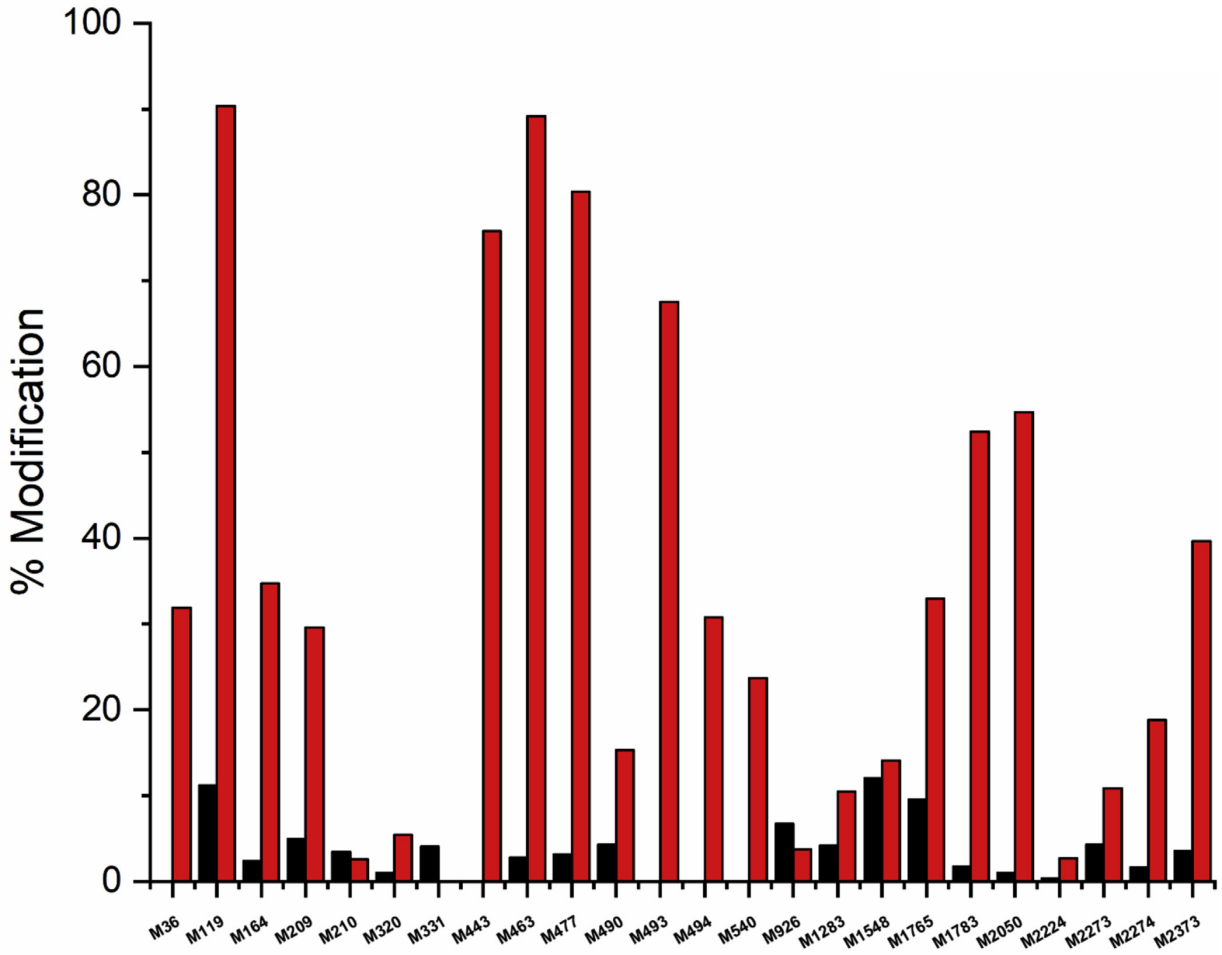
**Quantification of methionine (M) oxidation to the corresponding (*m/z* +16) sulfoxide in the monomeric fraction of FN (1 μM) after exposure to 50 μM ONOOH in buffer containg 150 mM NaCl, and subsequent separation using gel filtration chromatography (SEC) using 150 mM NaCl and 0.1% SDS**. *Horizontal axis* indicated residue number in the sequence and the *vertical axis* is the % modification at the particular site, with *red bars* indicating FN treated with ONOOH, and *black bars* indicating data from control protein. Where no bar is visible, the level of modification was below the detection limit, or the parent residue was not detected. Data from a single representative experiment are presented because of variation in the background (control) level of oxidation between experiments. FN, fibronectin; ONOOH, peroxynitrous acid and its conjugate anion.

As ONOOH is also known to target Met residues (25) and these are moderately abundant in FN (27 in the human isoform 1 sequence, Uniprot ID P02751), the number of oxidized Met was also investigated for both the control protein and the 50 μM ONOOH condition (with low salt buffer, *i.e.*, 150 mM NaCl). For the control protein, 19 of the 27 Met residues were identified as oxidized to a limited extent, whereas for the 50 μM ONOOH condition, 23 residues were identified as oxidized (Fig. 6). In the control samples, the extent of oxidation was <5% at all sites with the exceptions of Met119 (11.1%), Met926 (6.7%), Met1548 (12.1%), and Met1765 (9.5%). These modest levels of alteration may reflect either modifications present on the original protein (which is obtained from human plasma and hence may reflect *in vivo* modification) or from *ex vivo* protein handling and processing, including the sample preparation methods (a well-established problem in MS protocols; see *e.g.*, (38)). In contrast, the extent of modification in the ONOOH-treated samples was markedly elevated at nearly all sites, with the % modification values being in the range 10–90% at 19 of the residues. In each case, the modification was detected as a *m/z* +16 species, with this being assigned to the addition of a single oxygen atom and formation of the sulfoxide from Met.

### Mass spectrometric characterization of protein cross-links

Previous studies with multiple oxidants, including ONOOH and HOCl, have provided evidence for the formation of both reducible and nonreducible covalent cross-links between FN chains (33, 39, 40). The former links are likely to be disulfide bridges, whereas the nonreducible species have been identified, at least in part, as being because of di-Tyr linkages (33). Thus, higher mass species have been detected by SDS-PAGE, and di-Tyr was detected by both amino acid analysis and *via* the use of specific antibodies (33). However, whether di-Tyr is the sole contributor to the irreversible cross-links, whether it is intramolecular and/or intermolecular in nature, and the position of the residues involved within the FN sequence have not been established; these facets were therefore investigated.

**Figure 7:**
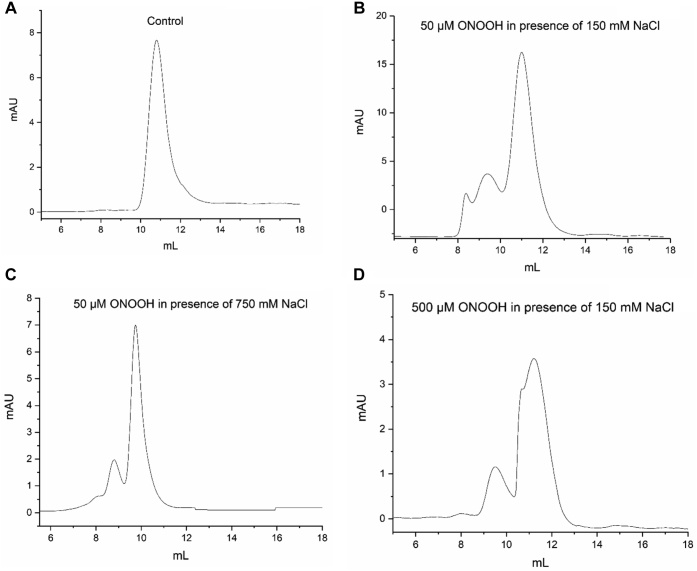
**Exposure to ONOOH impacts on the elution profile of FN subjected to gel filtration chromatography (SEC)**. Chromatograms are presented for 50 μg FN treated with 10 mM DTT and 1% SDS before SEC (denaturing conditions). *A*, FN without ONOOH treatment (control). *B*, FN treated with 50 μM ONOOH in the presence of 150 mM NaCl. *C*, FN treated with 50 μM ONOOH in the presence of 750 mM NaCl. *D*, FN treated with 500 μM ONOOH in the presence of 150 mM NaCl. Samples were separated in isocratic mode at a flow rate of 0.5 ml min^−1^ with 50 mM sodium phosphate buffer, containing 150 mM NaCl and 0.1% SDS, at pH 7.4, on a Superose 6 Increase 10/300 GL column. DTT, dithiothreitol; FN, fibronectin; ONOOH, peroxynitrous acid and its conjugate anion.

SEC was used to separate the parent and oxidized FN, as described above, into monomer and dimer fractions. The elution time of the former (∼10.7 min) was assigned by comparison with the nonoxidized parent protein pretreated with DTT/SDS (see Experimental procedures) before separation (Fig. 7*A*), whereas the elution time of the native dimer species (∼9.5 min) was determined by similar analyses but without the DTT treatment; in this case, a low amount (<10%) of monomer species was also detected (data not shown). As shown in Figure 7*B*, treatment of FN with 50 μM ONOOH, in the presence of 150 mM NaCl, and subsequent incubation with 10 mM DTT/1% SDS, and separation by SEC gave rise to two major peaks assigned to monomeric and dimeric chains. As the samples were pretreated with DTT before separation, any dimer species present after oxidation are likely to contain interchain cross-links, whereas any cross-links detected in the monomer fraction would be intramolecular in nature. Similar analyses carried out on samples treated with 50 μM ONOOH, in the presence of 750 mM NaCl (Fig. 7*C*), also provided evidence for the presence of both dimer and monomer species, though these fractions eluted at earlier time points than those separated under normal physiological salt (150 mM NaCl) conditions. Treatment of FN with 500 μM ONOOH, in the presence of 150 mM NaCl, provided evidence for more extensive modification and the presence of a heterogenous population of FN species, as judged by the detection of distorted peaks (Fig. 7*D*). The separated fractions were then subjected to LC-MS analysis for the presence of cross-links.

**Figure 8:**
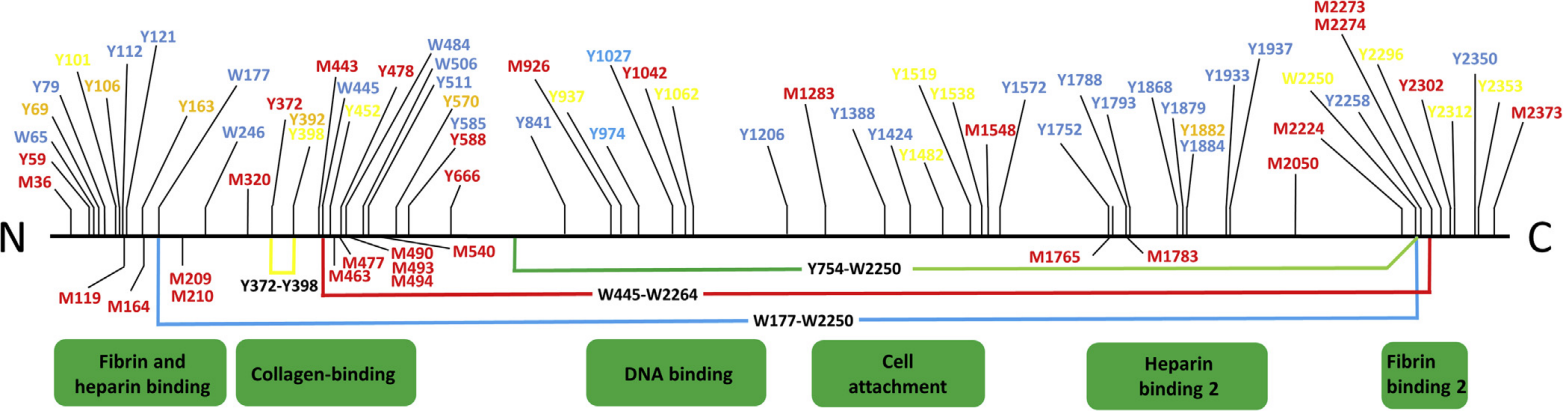
**Scheme illustrating the sequence positions of the amino acids (Tyr, Y; Trp, W; Met, M) that are modified (*thin lines* in top part of figure) and cross-links (*thick lines* in lower part of figure) in FN treated with ONOOH, and their relationship to the known functional domains of FN**. Tyr and Trp residues are color coded corresponding to the extent of modification for the 1 μM FN, 50 μM ONOOH, and 150 mM NaCl condition, with *red* corresponding to level of nitration > than 30%; *orange* corresponding to 20 to 30%; *yellow* corresponding to 10 to 20%; and *blue* corresponding to 1 to 10% nitration. Methionine (Met, M) residues indicated in *red* were detected as the oxidized (sulfoxide) species (+16). It should be noted that although this representation indicates that these linkages are intrachain for clarity, the experimental data is consistent with some of these being interchain or between dimers; see text for further details and discussion. FN, fibronectin; ONOOH, peroxynitrous acid and its conjugate anion.

The MS strategy, based on a H_2_^16^O *versus* H_2_^18^O labeling approach (41), yielded distinctive peaks differing by +4 Da for the H_2_^18^O digested species compared with the H_2_^16^O reactions for noncross-linked peptides (from incorporation of two ^18^O atoms at the new carboxyl terminus) and +8 Da for cross-linked peptides (due to the presence of two new carboxyl termini, each with two ^18^O atoms). In total, four cross-linked peptides were identified, including one di-Tyr cross-link, one Tyr-Trp cross-link, and two di-Trp cross-links (Table 2). Of these species, the di-Tyr cross-link (Tyr372-Tyr398) and one di-Trp linkage (Trp445-Trp2264) were identified with both NaCl conditions (150 mM and 750 mM), whereas the other two cross-linked peptides were only identified under the normal (150 mM) NaCl condition that corresponds to the compact conformation of the protein. This observation suggests that the regions of the FN structure that give rise to these species are likely to be in close proximity in the compact conformation but not the extended (see also Discussion). The Tyr372-Tyr398 cross-link was identified in FN exposed both to 50 μM and 500 μM ONOOH, whereas the other cross-links were only identified in FN exposed to 50 μM ONOOH. Comparison of the two SEC fractions (monomer *versus* dimer/oligomer) allowed information to be obtained as to whether these cross-links were intermolecular or intramolecular. Both the di-Trp cross-links and also the di-Tyr cross-link were identified in the dimer fraction from SEC, suggesting that these are intermolecular cross-links between the two monomers. The Tyr-Trp species, on the other hand, was only detected in the nonfractionated sample consistent with this being either intermolecular or intramolecular. A summary of the residues and positions of these cross-links is presented in Figure 8. These data indicate that *all* of the cross-links detected are present in two particular regions of the FN structure: between Y177-Y754 (*i.e.*, primarily within the collagen-binding domain) and W2250-W2264, in the fibrin binding-2 domain, suggesting that these regions exist in close proximity to each other in the compact conformation of FN (see also Discussion).

**Table 2:** Identified cross-linked peptides in FN subjected to ONOOH

Cross-linked peptide (module)	ONOOH (μM)	NaCl (mM)	*m/z*	Charge	Observed mass (Da)	Mass error (ppm)	Fragmentation method	Monomer	Dimer	In-solution	MS method
TF**Y**^372^SCTTEGR (FNII_1_) **Y**^398^SFCTDHTVLVQTR (FNII_1_)	50, 500	150, 750	589.870	5	2944.312	0.8	HCD, EThcD	-	**✓**	**✓**	Universal, Targeted
GE**W**^**177**^TCKPIAEK (FNI_3_) **W**^**2250**^CHDNGVNYKIGEKWDR (FNI_11_)	50	150	708.328	5	3536.599	1.1	HCD, EThcD	-	**✓**	-	Universal, Targeted
**W**^**445**^CGTTQNYDADQK (FNII_2_) **W**^**2264**^DRQGENGQMMSCTCLGNGK(FNI_11_)	50	150, 750	801.326	5	4001.59	7.49	HCD, EThcD	-	**✓**	-	Universal, Targeted
VE**Y**^**754**^ELSEEGDEPQYLDLPSTATSVNIPDLLPGR (FNIII_2_) **W**^**2250**^CHDNGVNYK (FNI_11_)	50	150	997.055	5	4980.235	6.42	HCD	-	-	**✓**	Universal

EThcD, electron transfer dissociation supplemented with higher-energy collisional dissociation; FN, fibronectin; HCD, higher-energy collisional dissociation; ONOOH, peroxynitrous acid and its conjugate anion.

Observed mass-to-charge ratio (m/z) and observed mass (Da) with errors in parts per million are indicated. Amino acids forming cross-links are highlighted in *bold*, with residue numbers referring to the mature protein. Tryptic peptides detected after in-solution digestion, as well as in the fractions (monomeric or dimer) separated using size-exclusion chromatography (SEC) are indicated. Residues that were also detected as nitrated species are underlined.

Annotated spectra are displayed in Figs. S3 and S4.

Comparison of the cross-link data with the nitration data (see above) shows that two of the heavily nitrated residues, Tyr372 and Trp2250, were also involved in cross-link formation, whereas some of the other residues involved in cross-link formation (*e.g.* Tyr754 and Trp2264) were not detected as heavily nitrated species. These data indicate that nitration and cross-link formation are competitive processes for some residues, but not with others.

The di-Tyr cross-link (Tyr372-Tyr398), identified in the dimeric fraction from SEC and also in in-solution samples, showed the expected (−2.01 Da) mass loss compared with the sum of the parent sequences for a direct cross-link, and the theoretical mass (2944.311) corresponds closely to the experimental mass (2944.312 Da, mass error, 0.8 ppm). The MS/MS spectra revealed multiple fragments retaining the cross-link site, including the b1, b2, b3, b7, b9, and b12 ions from the (longer) α peptide and b3, b5, b6, and b8 from the (shorter) β peptide (Figs. S2 and S3) The di-Trp cross-link, Trp177-Trp2250, detected in the SEC dimer fraction also showed the expected mass loss (−2.01 Da) compared with the sum of the parent sequences and the theoretical mass (3537.599; including nitration of Trp2264) corresponding closely to the experimental mass (3537.597 Da, mass error, 1.1 ppm). The di-Trp cross-link, Trp445-Trp2264, detected in SEC dimer fraction also revealed the expected mass loss (−2.01 Da) compared with the sum of the parent sequences and the theoretical mass (4001.56) matching closely the experimental mass (4001.59 Da, mass error, 7.49 ppm). The MS/MS spectrum of this species also demonstrated evidence for this cross-link, where multiple fragments were found to retain the cross-link site (Figs. S2 and S3). Finally, one Tyr-Trp cross-link was also identified, Tyr754-Trp2250, which was detected in the in-solution sample. This cross-link also shows the expected mass loss (−2.01 Da) compared with the sum of the parent sequences and the theoretical mass (4980.267), corresponding closely to the experimentally determined mass (4980.235 Da, mass error, 6.42 ppm).

### Mapping of nitrated and cross-linked species to the FN structure

**Figure 9:**
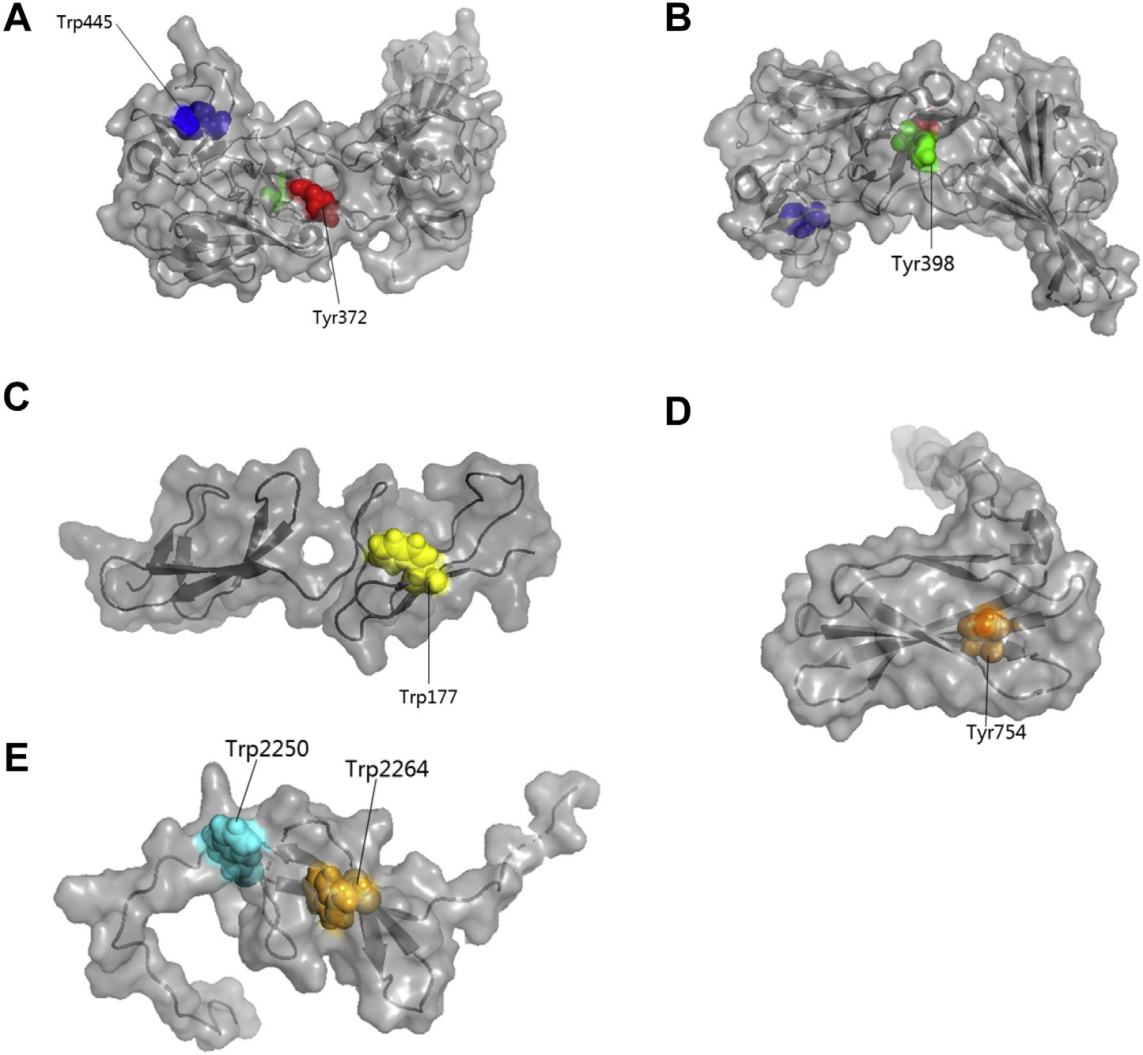
**Rendering of residues involved in cross-linking**. *A* and *B*, correspond to the structure of FNI_6_, FNII_1_, FNII_2_, FNI_7_, FNI_8_, FNI_9_ with accession number PDB 3m7p (52) (*B* is rotated vertically by 180°). *C*, shows the structure of FNI_2_, FNI_3_ with accession number PDB 2cku (63). *D*, shows the structure of FNIII_2_ with accession number PDB 2h41 (64). *E*, corresponds to the structure of FNI_11_ (PDB 2ec3; unpublished data deposited in PDB). PyMOL software was used to render the structures. FN, fibronectin.

A summary of all the modifications detected on FN is presented in Figure 8 and Fig. S4. The position of the nitrated residues and those involved in cross-linking were mapped to the available (partial) three-dimensional structures of the protein; no complete structure is available to date (Fig. 9 and Figs. S5–S14). As shown in Figure 9, the residues involved in all of the cross-links are surface accessible, consistent with the ability to form either intramoleccular or intermolecular linkages. Although Tyr372 and Tyr398 appear in relatively close proximity within the collagen-binding domain (Fig. 9, *A* and *B*), the cross-link involving these residues was only detected in the dimer fraction, indicating that these residues are part of an intermolecular (interchain) cross-link. All of the residues involved in the other cross-link pairs are remote from each other including the Tyr754 and Trp2250 residues detected as a cross-link in the in-solution sample and hence either involved in an intermolecular or a long-range intramolecular linkage. The structural implications of these cross-links for the quaternary organization of FN are expanded on in the Discussion.

Analysis of the sites of nitration and also the Tyr and Trp residues that were not detected as nitrated indicates that most of the modified residues are surface accessible in the available partial structures, suggesting a ready accessibility of these species to ONOOH (Figs. S5–S12). Furthermore, classification of the residues into approximate decile percentages of modification (>30%, 20–30%, 10–20%, and 1–10%) suggests that the most exposed Tyr and Trp residues and those with the highest levels of surface exposure were the most heavily modified (highlighted in red in the structures). Consistent with this crude classification, many of the residues with low or no nitration (highlighted in *blue* and *green*, respectively) were found to be more buried within the available structures and hence might be less accessible to the hydrophilic oxidant.

## Discussion

Current data indicate that FN is a key protein in the assembly of native basement membranes and other ECMs *via* its interactions with other ECM proteins (1, 4). It is also a key player in organismal responses to tissue injury with FN, and its interactions with fibrin, being essential components of blood clots and the temporary matrix created as sites of damage to restore homeostasis (1, 4). FN is also a key modulator of cell adhesion and behavior through its interactions with integrins and *via* other signaling pathways (7, 42, 43). Dysregulation of FN production and behavior has been associated with poor wound healing and disease including heart failure, cardiovascular syndromes, and many fibrotic pathologies (17, 19, 44, 45, 46). Evidence has been reported for the presence of modified FN in atherosclerotic lesions and heart failure (14, 19) and for a role for excess FN in the development of multiple diseases including atherosclerosis and fibrosis in multiple organs (17, 19, 44, 45, 46). Previous studies have shown that FN is susceptible to modification by multiple oxidant species generated at sites of inflammation, including within atherosclerotic lesions (33, 37, 39). These modifications appear to have functional consequences with reduced adhesion, a modulation of proliferation, and altered gene, protein, and cytokine expression by cells (*e.g.*, endothelial and smooth muscle cells) exposed to modified FN (33, 37, 39). Data have been reported for the overall extent of modification and effects on the structural integrity of FN exposed to ONOOH (and also another inflammatory oxidant, HOCl), but the exact nature of the modifications and cross-links, and their position within the FN sequence have not been investigated in detail (33, 37, 39). These modifications may serve as biomarkers of ongoing inflammation and damage in the artery wall, as turnover of modified ECM is likely to result in the release of the modified materials, as peptides or free amino acids, into plasma and urine. In this regard, it is of interest to note that elevated levels of some of these species, including cross-linked materials such as di-tyrosine, have been detected in such fluids in response to inflammatory and oxidative insults (*e.g.*, sepsis, exercise, smoking, interstitial lung disease and fibrosis (47, 48, 49, 50)), though their exact source (*i.e.*, from which tissue and protein they are derived) has yet to be fully identified.

In the studies reported here, we show that exposure of FN (final protein concentration 1 μM) to 50 or 500 μM ONOOH (*i.e.*, a 50-fold or 500-fold molar excess of ONOOH) results in widespread modification of Tyr, Trp, and Met residues, as well as the formation of intrachain and interchain cross-links. It is shown, with the 50-fold molar excess of ONOOH, that many, but not all, of the Met residues present in FN are converted to sulfoxides in high yield, and that Tyr residues are typically modified to a much greater extent (both in terms of the % modified of the total number, and the extent of modification at particular sites) than Trp residues. The extent of Met oxidation to the sulfoxide and Tyr and Trp nitration varies dramatically across the FN sequence. For Met oxidation, some of the residues are modified in a near quantitative manner, whereas others are not modified significantly (Fig. 6); this may be due, at least in part, to residue accessibility (38). For Tyr nitration, some residues are also modified to a high extent (up to ∼35%), whereas others are not detected with any modifications. A similar pattern is seen with Trp residues, with a very small number being highly modified (up to 15% at Trp2250), but others show little or no alteration.

Interestingly, with higher levels of oxidant (500-fold), the extents of nitration at particular residues do not appear to increase in a linear manner. Thus, the increase in extent of modification at modestly modified sites (with a 50-fold molar excess of ONOOH) are enhanced to a much greater extent than at those that are heavily modified at low molar excesses. The reason for this is not currently clear, but it may be because of changes in protein structure that either expose previously buried or poorly exposed residues making these more accessible to oxidant modification or structural changes that hinder further modification at the initially damaged residues. This hypothesis is supported by the studies carried out with FN modified in both normal (150 mM) and high (750 mM) NaCl-containing solutions; this alteration in salt concentration is known to result in a change in FN conformation from a condensed to extended structure (3, 8). These salt-induced changes may also mimic, in part, the unfolding that occurs during blood clot formation (for the plasma isoform) and fibril formation for the cell-derived species. The presence of such extended structures is shown to result in an altered pattern of nitration, with some residues having a decreased, and others a markedly increased extent of modification, though the *overall* level of modification is not markedly affected. These changes indicate that the protein conformation has a dramatic effect on the extent of alteration at a particular residue and also the sites of modification induced by ONOOH (and possibly other oxidants as well).

In addition to the extensive nitration of Tyr and Trp residues and oxidation of Met residues, four sets of cross-links were detected with three of these involving interchain linkages (as evidenced by their detection in the dimeric fraction obtained from SEC) and one either intrachain or interchain bond. A previous study has identified di-Tyr as being present in ONOOH-modified FN (33), but we show here that this is only one of the cross-links formed, with both di-Trp and Trp-Tyr linkages also observed. This probably rationalizes the low levels of di-Tyr detected previously (33), and the contrasting detection of significant levels of cross-linked materials on SDS-PAGE gels (33). It is shown that these cross-links are localized to particular domains on FN. This is proposed to be because of an association of specific regions in the compact conformation of FN, as these cross-links are formed from the dimerization of short-lived radicals (Tyr phenoxyl and Trp indolyl) which would require a close spatial proximity between the relevant sites.

**Figure 10:**
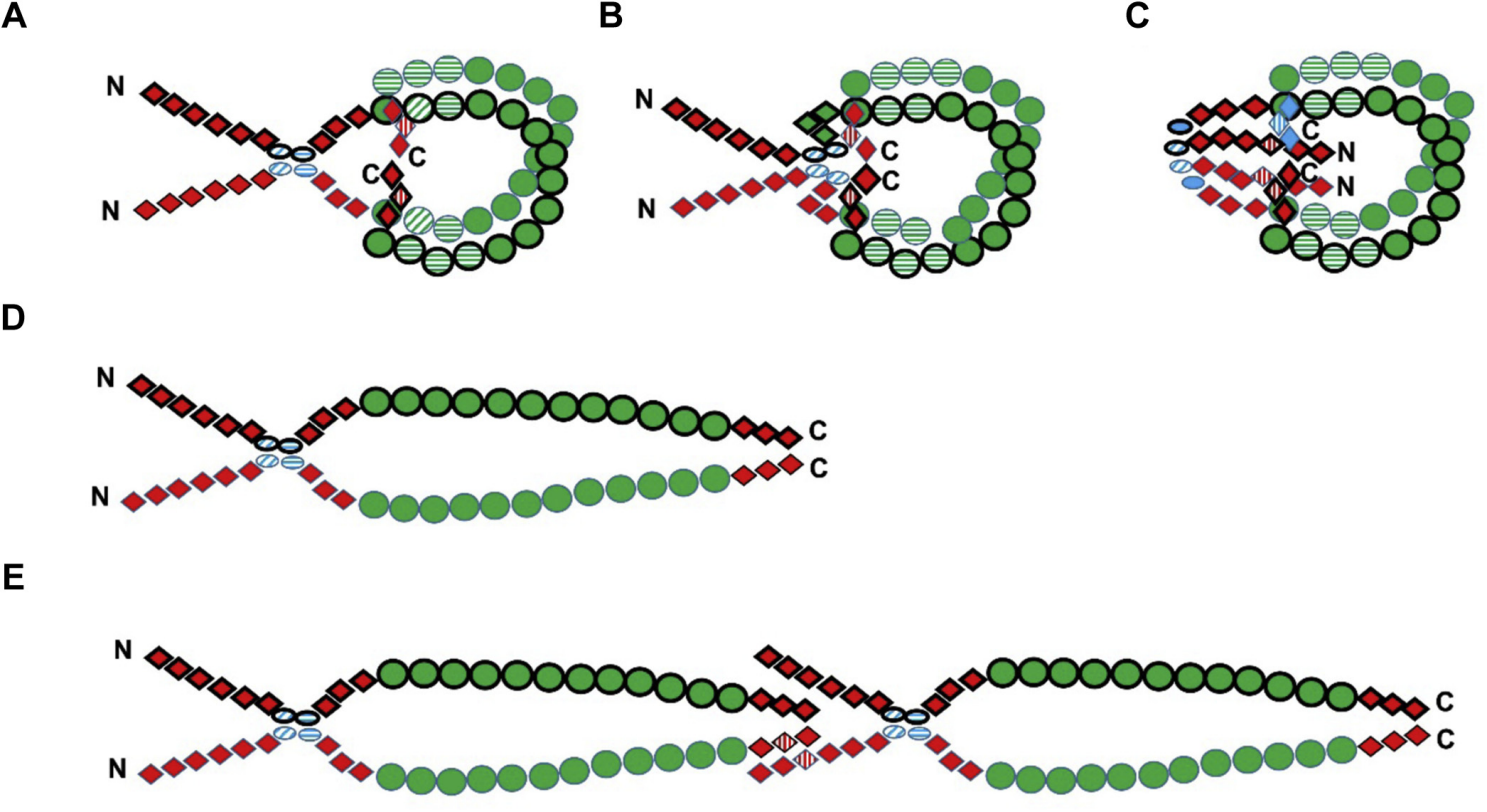
**Proposed FN conformations of compact and extended FN taking into account cross-linking and previous data on electrostatic interactions between modules** (8). Two FN monomers connected by C-terminal crosslinks are shown with and without *bold outline*. FN I, II, and III modules are displayed as *red diamonds*, *blue ovals*, and *green circles*, respectively. Modules involved in either cross-linking or electrostatic interactions are shown with *horizontal* and *vertical stripes*, respectively. Modules involved in both cross-linking and electrostatic interactions are shown with diagonal stripes. Models of the compact conformation with cross-links between (*A*) W2250 (FNI_11_)-Y754 (FNIII_2_) and Y372 (FNII_1_)-Y398 (FNII_1_); (*B*) W2264 (FNI_11_)-W445 (FNIII_2_) and Y372 (FNII_1_)-Y398 (FNII_1_); (*C*) W2250 (FNI_11_)-W177 (FNI_3_) and Y372 (FNII_1_)-Y398 (FNII_1_). Models of the extended conformation with cross-links between (*D*) Y372 (FNII_1_)-Y398 (FNII_1_); (*E*) Y372 (FNII_1_)-Y398 (FNII_1_) and W177 (FNI_3_)-W2250 (FNI_11_). FN, fibronectin.

The majority of these cross-links are interchain and connect regions near the N-terminus and C-terminus of the FN polypeptide. Because the monomer chains are constrained by the pair of disulfide bonds near the C-termini, these data are interpreted in terms of “folded back” or “coiled” conformations, in agreement with previous models (3) and the known communication between the cell-binding and heparin-binding 2 region, with regard to the control of cell adhesion and integrin binding (51). Two of the four cross-links were also detected in the extended conformation of FN suggesting that these are linking regions that are in close proximity in both the compact and the extended conformation of the protein. Electron microscopy data clearly demonstrate that plasma FN in the compact conformation has a rather flexible structure that can adopt multiple different conformations in solution (3). It cannot therefore be assumed that all the cross-links identified in the present study are derived from a homogenous population of molecules in a single well-defined conformation. It seems more reasonable to consider the identified cross-links as representations of subsets of FN molecules with different quaternary structures. Accordingly, we propose a set of models that accommodate interactions between the cross-linked species in different types the compact conformations (Fig. 10). The models we propose accommodate cross-links between FNI_11_ of one monomer and FNI_3_, FNII_2_, or FNIII_2_ of the other monomer, as well as intermolecular cross-links between FNII_1_ modules. These models also take into consideration electrostatic interactions between FNIII_12–14_ and FNIII_2–3_ modules from the two polypeptide chains as proposed previously (8). The current model can also be adopted to match intermolecular interactions between FNII_1_ modules in the extended conformation (Fig. 10). The observed intermolecular cross-links between FNI_11_ and FNI_3_ in the extended conformation is however difficult to rationalize in terms of interactions between the two polypeptide chains of the FN dimer. We therefore propose that such links may be formed between chains derived from two different FN dimers in close association. One major caveat on these models is that the data on which they are based are only from the plasma isoform, and whether similar features are generated from cellular FN, which contains the additional EDA/EDB, is unclear.

It is also interesting to note that several of the residues involved in the identified cross-links show markedly different nitration levels in the low and high salt concentrations corresponding to the compact and extended confirmation, respectively. In particular, Trp2250, stabilizing both cross-links that were identified exclusively in the compact conformation, was nitrated to a much higher level in the high salt buffer representing the extended conformation. This makes sense, because this residue is expected to be less accessible in the compact conformation where Trp2250 is cross-linked to either Trp177 or Tyr754. Furthermore, Tyr398 which forms a cross-link with Tyr372 in both the compact and the extended conformations was not identified as nitrated in neither of these conditions, again consistent with poor accessibility of the cross-linked residue. It is remarkable that three of the four cross-links identified here involved the closely spaced Trp2250 and Trp2264 in the FNI_11_ module of the fibrin-binding 2 domain (Fig. 8). These results suggest that this region is important in stabilizing intermodule interactions of FN. The two cross-links that were identified in both the compact and extended conformations (Tyr372-Tyr398 and Trp445-Trp2264) involve residues from the two FNII modules in the collagen-binding domain. Interestingly, these modules have previously been shown to form intermolecular dimer contacts in the crystal structure of FNI_6_FNII_1,2_FNI_7,8,9_ (52).

The localization of the nitration sites to particular Tyr and Trp residues is in accord with a more limited previous study, where 24 Tyr and four Trp residues were identified as modified with a high (500-fold) molar excess of ONOOH (33). Comparison of the sites detected in the current study with this previous data (obtained from MS analysis of peptides arising from specific gel bands) indicates that 19 out of the 24 Tyr residues identified as nitrated in this previous research were also detected as nitrated in this work, whereas none of the four Trp residues detected as nitrated in the previous work (33) were found to be nitrated in the current study. The latter difference may arise from the low levels of nitration detected on the Trp residues, unlike the higher levels of modification detected with many of the Tyr residues.

**Figure 11:**
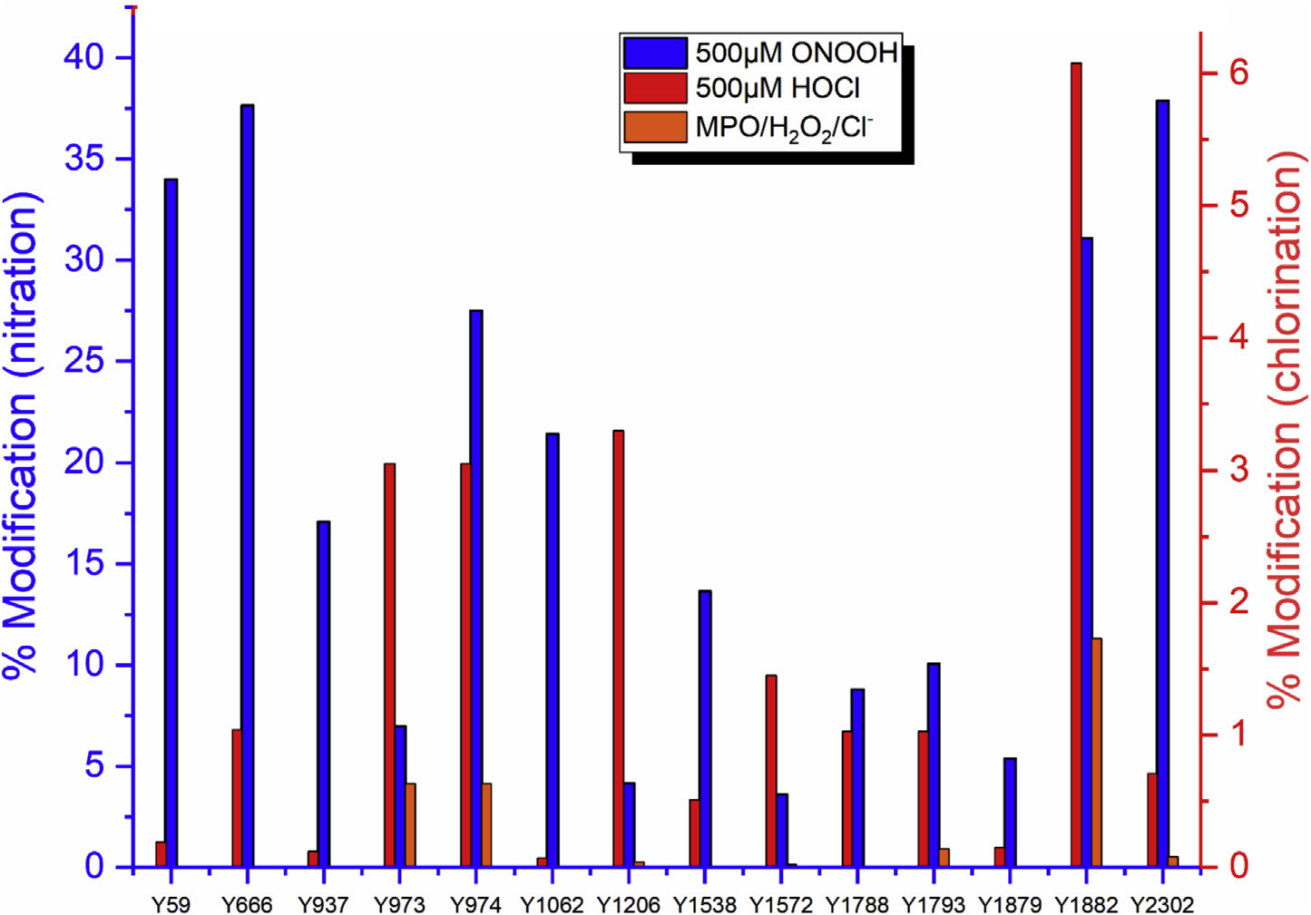
**Comparison of the extent of modification (nitration or chlorination) at Tyr residues in FN exposed to 500 μM ONOOH (*blue bars*) or 500 μM HOCl (*red bars*) or MPO/H**_**2**_**O**_**2**_**/Cl**^**−**^**system with 500 μM H**_**2**_**O**_**2**_**(*orange bars*); data from** (37). FN, fibronectin; HOCl, the physiological mixture of hypochlorous acid and its conjugate anion ^−^OCl; MPO, myeloperoxidase; ONOOH, peroxynitrous acid and its conjugate anion.

FN can also be modified by oxidants generated by the heme enzyme MPO, which generates both HOCl and a number of other one-electron (radical) and two-electron oxidizing species (37, 53). Differences were detected between the extents, location, and types of modifications detected with reagent HOCl and an MPO/H_2_O_2_/Cl^−^ system supplied with an equal concentration of H_2_O_2_ to give a similar total oxidant yield (37). These differences have been proposed to be because of binding of the enzyme to FN (53), which might direct oxidation to specific sites close to the active site of MPO. As such effects would not complicate the analysis of the current nitration data, using reagent ONOOH, it was of interest to compare the pattern and extent of nitration at different sites *versus* chlorination (and also other sites of modification). This has been carried out for 500 μM ONOOH *versus* 500 μM reagent HOCl and also 500 μM ONOOH *versus* the modifications induced by the MPO/H_2_O_2_/Cl^−^ system with 500 μM H_2_O_2_ (Fig. 11).

The nitration levels arising from treatment with ONOOH were significantly higher than the corresponding levels of chlorination detected with HOCl, which may be due, at least in part, to differences in the overall reactivity of ONOOH *versus* HOCl. Thus, HOCl is more reactive with many amino acid sidechains than ONOOH, but reacts only slowly with Tyr (*k* 44 M^−1^ s^−1^ for HOCl; a corresponding value for ONOOH is difficult to define as this involves two consecutive radical reactions (25, 54, 55, 56, 57)). In contrast, ONOOH reacts with only modest rate constants with most sidechains, with reaction with Tyr therefore likely to be more quantitatively important. However, it is of interest to note that a significant number of Tyr residues were modified by *both* oxidants, indicating that these two species share some common targets, possibly as a result of a high degree of surface exposure or local environmental factors that make particular residues highly reactive. Furthermore, the Tyr residue showing the highest level of chlorination, Tyr1882 (6.1% chlorination) was also found to have a high (though not the highest, *cf.* Figs. 2 and 11) level of nitration (26.9%); Fig. S13 shows that this residue has a very high surface exposure. However, other Tyr residues with a high level of chlorination (*e.g.*, Tyr973 and Tyr1206) were not detected as being heavily nitrated (Fig. 11) and do not appear to have a high surface exposure (Fig. S11), indicating that surface exposure is unlikely to be the only determining factor. A similar analysis is not possible for Trp, as HOCl does not give significant levels of chlorination on this residue, but rather oxidation products (58). In general, more Tyr residues were detected as nitrated (45 in total) compared with the identified chlorinated Tyr (17 in total). All Tyr detected as chlorinated were also identified as nitrated in this study, except three: Tyr687, Tyr941, and Tyr2076, which were only detected as chlorinated. Only three Met residues were detected as oxidized after treatment with HOCl (Met926, Met1783, and Met2050). These three residues were also detected as oxidized in the current study after treatment with 50 μM ONOOH but to very different extents (marked oxidation at Met1783 and Met2050, but very modest oxidation at Met 926) consistent with a different pattern of reactivity between ONOOH and HOCl, with 20 other Met resides also detected as being oxidized by ONOOH, and some to a very high extent.

As shown in Figure 11, the level of Tyr chlorination after treatment with the MPO/H_2_O_2_/Cl^−^ system using 500 μM H_2_O_2_ was quite low compared with the level of nitration detected on treatment with 500 μM ONOOH. The Tyr residue with the highest level of chlorination after treatment with the MPO system, Tyr1882 (1.7% chlorination), was also found to have a high level of nitration. Overall, fewer Tyr were found to be chlorinated with the MPO system (8 Tyr residues) compared with the nitrated Tyr residues, and many more Tyr residues appear to be nitrated by 500 μM ONOOH, than chlorinated by the same concentration of HOCl, or the MPO/H_2_O_2_/Cl^−^ system with 500 μM H_2_O_2_.

A significant number of the observed site of Met oxidation and Tyr and Trp nitration occur within the known functional domains of FN (*cf.* annotations on Fig. 8) suggesting that these alterations even if present at low levels, individually, on single molecules of FN may have an overall cumulative biological effect both on ECM assembly (as suggested by the modifications in the domains associated with this activity) and also on cell attachment and binding. The latter is consistent with the reduced adherence and spreading of naïve (no oxidant exposure) endothelial cells on FN previously modified by ONOOH (33).

## Experimental procedures

### Reagents and proteins

All chemicals including lyophilized human plasma FN (catalog number: F1056) were purchased from Sigma-Aldrich unless stated otherwise. ^18^O water (95.7% pure) was purchased from Sercon. ONOOH was synthesized in a two-phase system using isoamyl nitrite/H_2_O_2_, with residual H_2_O_2_ removed by treatment with MnO_2_ (59). Stock solutions of ONOOH were stored at −80 °C and used immediately after defrosting. ONOOH concentrations were determined spectrophotometrically at 302 nm before use, based on its extinction coefficient (60). Stock solutions of oxidant were diluted in 0.1 M NaOH before use, with low volumes added to strongly buffered PBS (100 mM sodium phosphate, pH 7.4) mixtures to minimize pH changes.

### Oxidation of human plasma fibronectin

FN (180 μl of a 1 mg ml^−1^ stock) was diluted with sodium phosphate buffer (final concentration 100 mM), pH 7.4, containing NaCl at either 150 or 750 mM (to give a final protein concentration of 1 μM) and exposed to ONOOH (50 μM and 500 μM) for 20 min at 21 °C. Under these conditions, complete oxidant consumption occurs because of the high reactivity and hence short half-life of this species (25).

### Separation of native fibronectin by gel filtration chromatography

Native FN was equilibrated for 1 h at 21 °C with PBS, pH 7.4, containing either 150 mM or 750 mM NaCl and separated by SEC using a Superose 6 Increase 10/300 GL column (GE Healthcare) on an Äkta purifier (GE Healthcare) equilibrated with 50 mM sodium phosphate buffer (pH 7.4) containing either 150 mM or 750 mM NaCl in isocratic mode at 0.5 ml min^−1^.

### Separation of denatured fibronectin by gel filtration chromatography

For fractionation before MS analysis, 180 μl FN (1 mg ml^−1^ stock) exposed to either 50 or 500 μM ONOOH (in a total volume of 675 μl) was reduced using 75 μl 200 mM DTT and 750 μl 2% (v/v) SDS added, followed by incubation at 70 °C for 10 min. Subsequently, 93.7 μl of 400 mM iodoacetamide was added, followed by incubation for 30 min in the dark. The samples were then concentrated to approximately 200 μl with Vivaspin 30 kDa filters, and 100 μl was loaded on to a Superose 6 Increase 10/300 GL column and separated in isocratic mode at a flow rate of 0.5 ml min^−1^ with 50 mM sodium phosphate buffer containing 150 mM NaCl and 0.1% SDS at pH 7.4.

### Protein digestion

Unfractionated FN samples were subjected to in-solution trypsin digestion. Fractions (1.5 ml) from gel filtration chromatography (see above) were collected and subjected to a filter-aided sample preparation protocol, using Amicon Ultra 0.5 ml centrifugal filters (10,000 Da cutoff). Two hundred microliter from each fraction was mixed with 200 μl 8 M urea in 0.1 M Tris buffer, pH 8.0, in the filter unit and centrifuged at 14,000*g* for 15 min. Four hundred microliter of 8 M urea in 0.1 M Tris, pH 8.0, was then added to the filter unit and centrifuged at 14,000*g* for 15 min, with this step repeated three times. Three hundred microliter 2 M urea in 0.1 M Tris (pH 7.0) was then added, followed by centrifugation at 14,000*g* for 10 min. Then, 200 μl of H_2_^16^O in 1.6 M urea (in 100 mM Tris, pH 7) was added, followed by centrifugation (as above). One hundred microliter H_2_^16^O 1.6 M urea (in 100 mM Tris, pH 7) together with 2 μl 0.1 μg μl^−1^ trypsin was then added to the filter units and incubated for 24 h at 21 °C. For analysis of cross-linked peptides, a parallel set of samples were processed where H_2_^16^O was replaced by H_2_^18^O. The filter units were then transferred to new collection tubes and centrifuged for 10 min at 14,000*g*, with the flow through collected. A further 50 μl of 0.5 M NaCl was then added, followed by centrifugation (as above) with the flow through collected and added to the previous fraction. The tryptic peptides were then subjected to StageTip solid-phase extraction on activated Empore C18 reversed-phase discs (3M), with the samples eluted using 10 μl 60% acetonitrile and 0.1% trifluoroacetic acid in water. The eluents were then dried down (Speedvac concentrator, 3 min), and samples were re-suspended in either 10 μl H_2_O or 10 μl H_2_^18^O and mixed in 1:1 ratio.

### Mass spectrometric analysis of modification sites

Peptides were separated and analyzed on an EASY nLC 1000 chromatograph (Thermo Fischer Scientific) equipped with a Pepmap EASYSpray column (Thermo Fischer Scientific; 3 μm, C18, 15 cm × 75 μm) coupled to an Orbitrap Fusion Lumos mass spectrometer (Thermo Fischer Scientific). Separation was performed at a flow rate of 250 nl min^−1^ using a gradient of solvents A (0.1% formic acid in water) and B (80% acetonitrile and 0.1% formic acid in water). Database searches were performed using MaxQuant (version 1.6.1.0) (61) with semispecific tryptic constraints, two missed cleavages, 1% peptide and protein level false discovery rates, and carbamidomethylation of Cys as a fixed modification. The variable modifications examined were +44.98 Da (Tyr, Trp), corresponding to 3-nitroTyr and 6-nitrotryptophan, and +16 Da (Met), corresponding to Met oxidation. The main search peptide tolerance was 4.5 ppm, and MS/MS tolerance was 20 ppm. The search was first run against a human proteome database from UniProt (created on 08–07–2018) containing ∼20,000 putative human proteins to evaluate additional contaminating peptides. Subsequent database searches were focused on the sequence of human fibronectin isoform 1 (FN1; Uniprot accession number: P02751), with the output from the latter used for further analysis. The percent modification at a particular site was estimated based on label-free quantification of the total spectra MS1 area ratios of the modified peptides divided by the sum of all the modified and corresponding nonmodified peptides detected for a specific sequence, determined using MaxQuant (61) and Skyline (62). Peaks with abundance changes of >10% were evaluated with respect to elution times, isotopic distribution, and MS/MS profiles using Skyline. Detailed information on the identified peptides is provided in the supplementary data.

### Mass spectrometric analysis of cross-linked peptides

Tryptic peptides digested separately in H_2_^18^O or H_2_^16^O were mixed 1:1 and immediately separated and analyzed on an EASY nLC 1000 chromatographic system coupled to an Orbitrap Fusion Lumos mass spectrometer (Thermo Fisher Scientific). Data were acquired either with a universal method characterized by a full MS Orbitrap scan followed by data-dependent high-energy collisional dissociation MS/MS scans, or a data-dependent method where a group of signals displaying mass shifts of +4, +6, and +8 Da were selected for MS/MS. In addition, data were also acquired with electron transfer dissociation supplemented with higher-energy collisional dissociation MS/MS scans, essentially as described previously (41). To identify cross-linked peptides, MS/MS data from H_2_^16^O labeled samples were subjected to database searches against the sequence of human fibronectin isoform 1 (FN1; Uniprot accession number P02751) using the software MassAI (Univ. of Southern Denmark, version July 2018). Raw data were converted to .mgf files using Proteome Discoverer version 1.4 (Thermo Fisher Scientific), and signals with an intensity below 2000 were subsequently filtered out in MassAI. The following database search settings were applied: fixed (carbamidomethylation of Cys) and variable (Met, +16 Da; Tyr and Trp, +44.98 Da) modifications; maximum two missed tryptic cleavages; parent mass tolerance 10 ppm; MS/MS peak tolerance 0.02 *m/z*. Di-tyrosine (Tyr-Tyr), di-tryptophan (Trp-Trp), and tyrosine-tryptophan (Tyr-Trp) were selected as potential cross-links. Cross-links with a score above 50 were manually validated based on fragment ion coverage and spectral quality and only cross-linked peptides that were consistently identified in replicates were accepted.

## Data availability

Raw mass spectrometry data files, and annotated mass spectra of modified peptides, is available in the MassIVE repository (project ID: MSV000086601; https://doi.org/10.25345/C5W509).

## Conflict of interests

The authors declare that they have no conflicts of interest with the contents of this article.
