# Supplementary material for: Cross-linking and modification of fibronectin by peroxynitrous acid: Mapping and quantification of damage provides a new model for domain interactions
Source: J Biol Chem. 2021 Feb 2;296:100360. doi: 10.1016/j.jbc.2021.100360 (PMC7950325; doi:10.1016/j.jbc.2021.100360)
Supplement: Supplemental Figures S1–S14 [file mmc1.pdf]

Cross-linking and modification of fibronectin by peroxynitrous acid: mapping and quantification of damage provides a new model for domain interactions

Michele Mariotti[1], Adelina Rogowska-Wrzesinska[2], Per Hägglund[1*], Michael J. Davies[1*]

[1]Department of Biomedical Sciences, Panum Institute, University of Copenhagen, Copenhagen, Denmark

[2] Department of Biochemistry and Molecular Biology and VILLUM Center for Bioanalytical Sciences, University of Southern Denmark, Odense, Denmark

* Joint senior and corresponding authors: M.J. Davies (davies@sund.ku.dk), P. Hägglund (pmh@sund.ku.dk)

Running title: Modification and cross-linking of fibronectin

SUPPLEMENTARY DATA

Keywords: Fibronectin, extracellular matrix, cross-links, nitration, oxidation, peroxynitrous acid, di-tyrosine, 3-nitrotyrosine, 6-nitrotryptophan

A

B

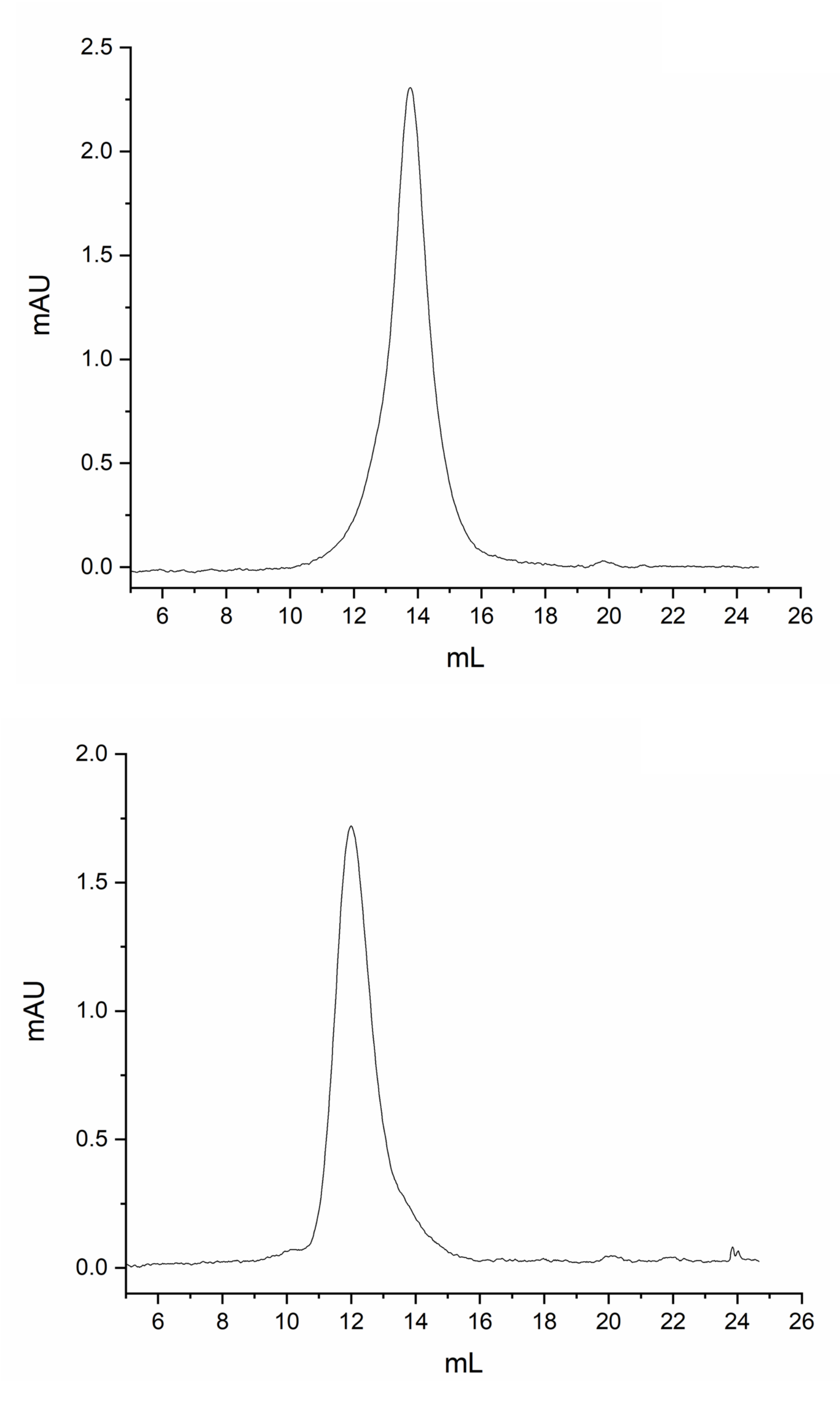

Supplementary Figure 1. SEC of 50 µg native FN in 150 mM (A) and 750 mM (B) NaCl-containing buffer.

A)

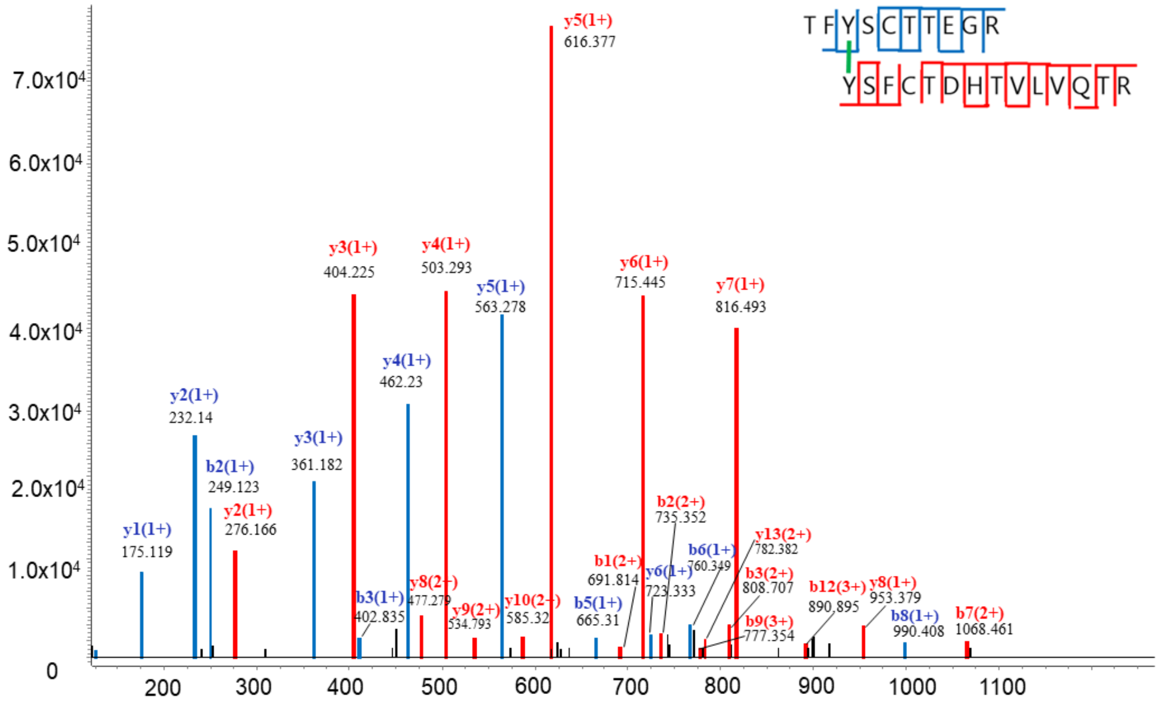

TFYSCTTEGR
YSFCTDHTVLVQTR
7.0x10^4
6.0x10^4
5.0x10^4
4.0x10^4
3.0x10^4
2.0x10^4
1.0x10^4
0
200
300
400
500
600
700
800
900
1000
1100
y5(1+) 616.377
y3(1+) 404.225
y4(1+) 503.293
y5(1+) 563.278
y6(1+) 715.445
y7(1+) 816.493
y4(1+) 462.23
y2(1+) 232.14
y3(1+) 361.182
b2(1+) 249.123
y2(1+) 276.166
y1(1+) 175.119
b2(2+) 735.352
y13(2+) 782.382
b6(1+) 760.349
b1(2+) 691.814
y6(1+) 723.333
b3(2+) 808.707
b3(1+) 402.835
y8(2+) 477.279
y9(2+) 534.793
y10(2+) 585.32
b5(1+) 665.31
b9(3+) 777.354
b12(3+) 890.895
y8(1+) 953.379
b8(1+) 990.408
b7(2+) 1068.461

B)

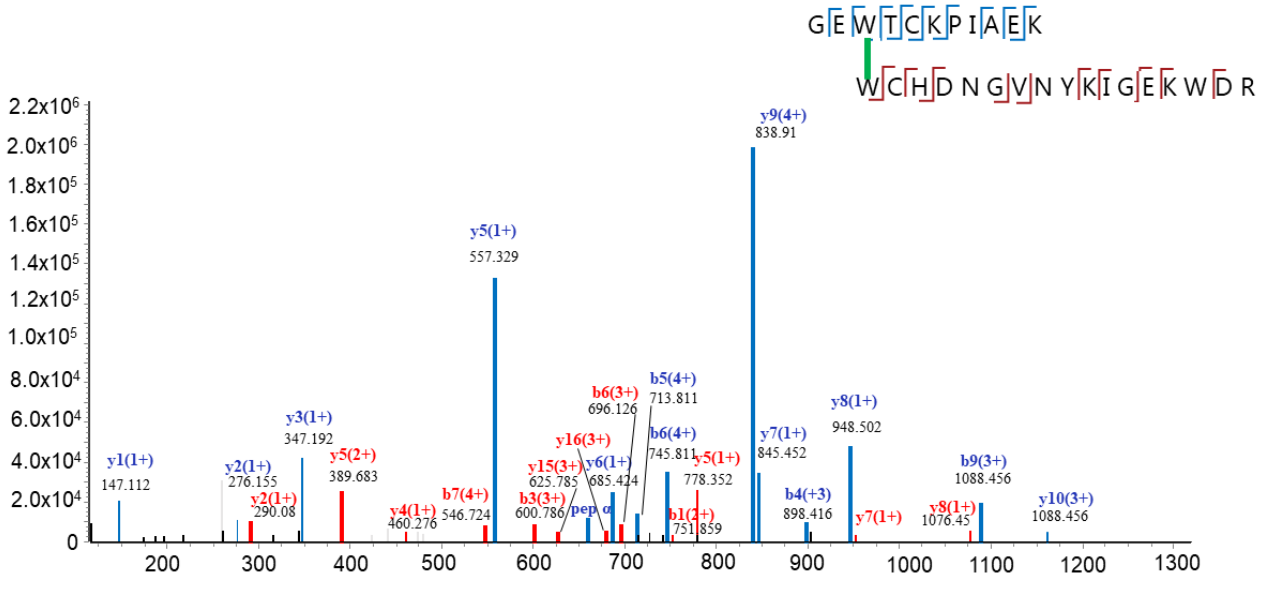

GEWTCKPIAEK
WCHDNGVNYKIGEKWDR
2.2x10^6
2.0x10^6
1.8x10^5
1.6x10^5
1.4x10^5
1.2x10^5
1.0x10^5
8.0x10^4
6.0x10^4
4.0x10^4
2.0x10^4
0
200
300
400
500
600
700
800
900
1000
1100
1200
1300
y9(4+) 838.91
y5(1+) 557.329
b6(3+) 696.126
b5(4+) 713.811
y16(3+)
b6(4+) 745.811
y15(3+) 625.785
y6(1+) 685.424
y5(1+) 778.352
y7(1+) 845.452
y8(1+) 948.502
y3(1+) 347.192
y1(1+) 147.112
y2(1+) 276.155
y5(2+) 389.683
y2(1+) 290.08
y4(1+) 460.276
b7(4+) 546.724
b3(3+) 600.786
pep α
b1(2+) 751.859
b4(+3) 898.416
y7(1+)
b9(3+) 1088.456
y8(1+) 1076.45
y10(3+) 1088.456

C)

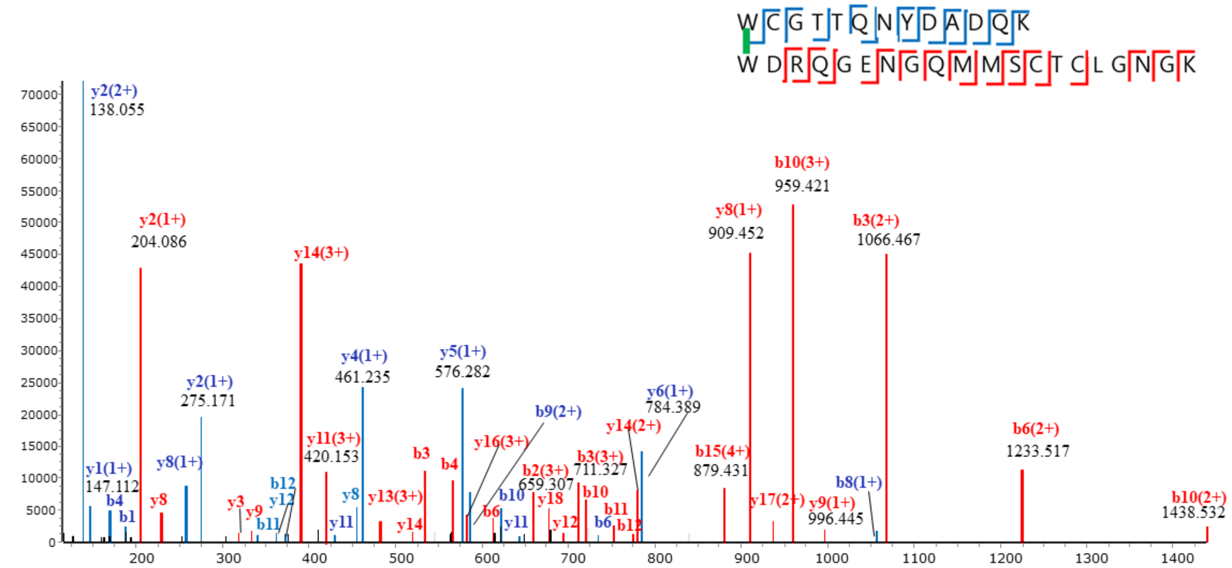

D)

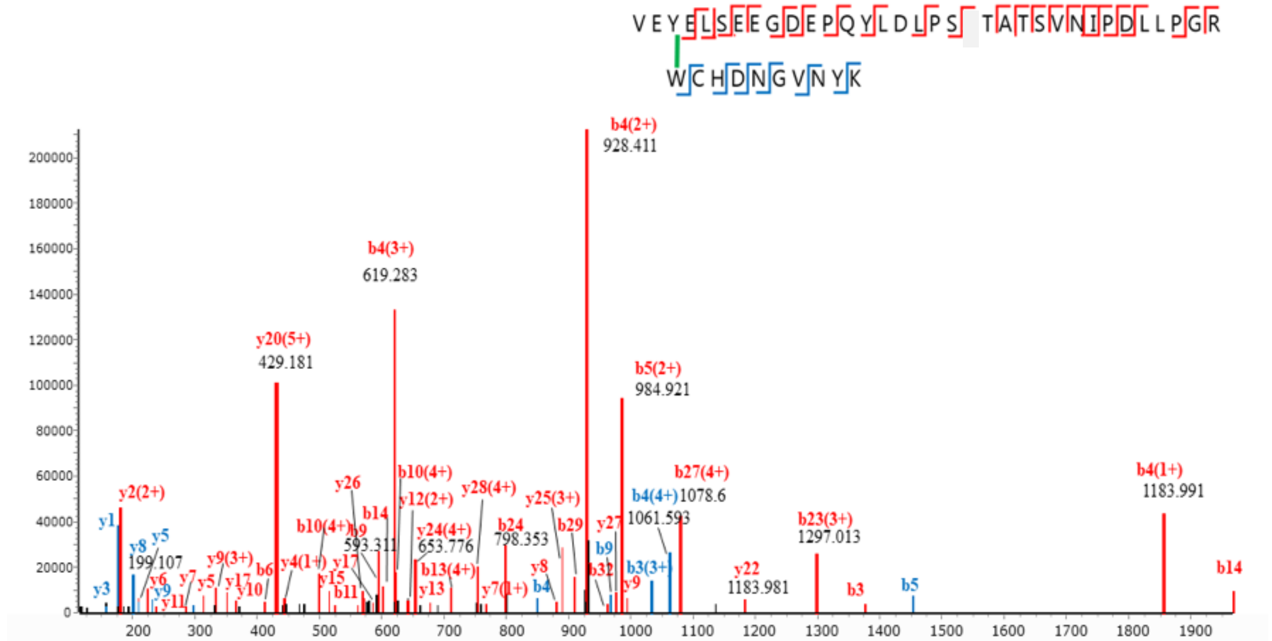

Supplementary Figure 2. HCD tandem mass spectra of cross-linked peptides. A) Spectrum of the quintuply-charged di-Tyr cross-linked peptide (TF**Y**^{372}SCTTEGR) (**Y**^{398}SFCTDHTVLVQTR), with precursor ion of *m/z* 589.87. B) Spectrum of the quintuply-charged di-Tyr cross-linked peptide (GE**W**^{177}TCKPIAEK) (**W**^{2250}CHDNGVNYKIGEKWDR), with nitration of **W**^{2264} matching the precursor ion of *m/z* 708.527. C) Spectrum of the di-Trp cross-linked peptide (**W**^{445}CGTTQNYDADQK) (**W**^{2264}DRQGENGQMMSCTCLGNGK), with precursor ion having *m/z* 801.326. D) Spectrum of the Tyr-Trp cross-linked peptide (VE**Y**^{754}ELSEEGDEPQYLDLPSTATSVNIPDLLPGR) (**W**^{2250}CHDNGVNYK), with the precursor ion of *m/z* 997.005.

Blue y and b fragments correspond to the α (shorter) peptide, while red y and b fragments correspond to the β (longer) peptide.

A)

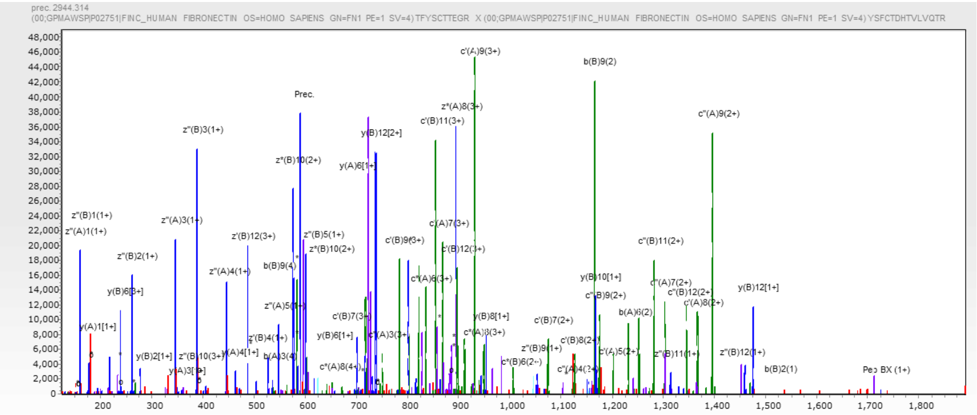

B)

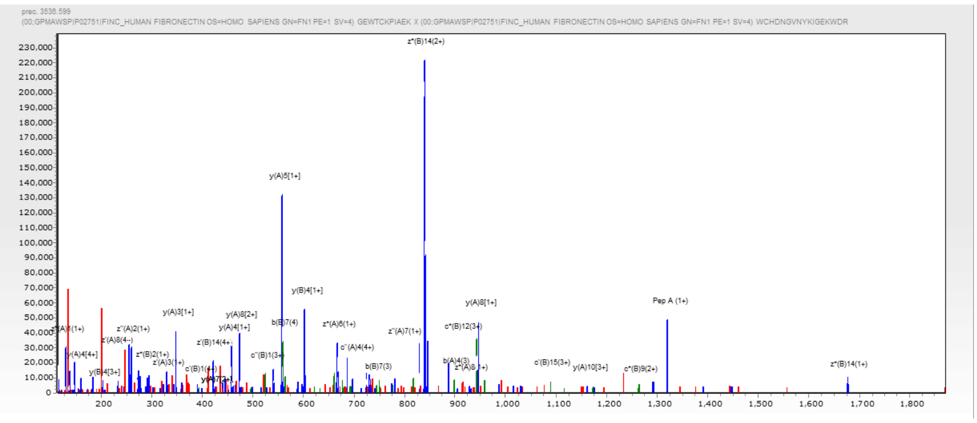

C)

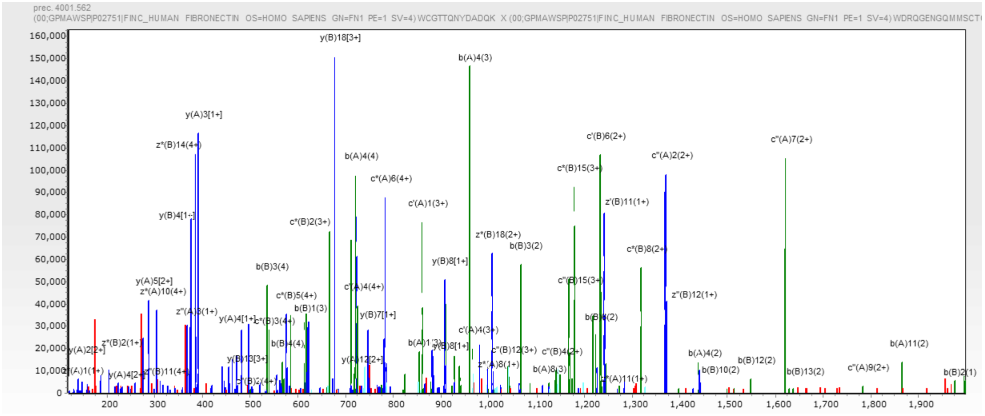

Supplementary Figure 3. EtHCD spectra of cross-linked peptides. A) Spectrum of the di-Tyr cross-linked peptide (TF**Y**^{372}SCTTEGR) (**Y**^{398}SFCTDHTVLVQTR). B) Spectrum of the di-Tyr cross-linked peptide (GE**W**^{177}TCKPIAEK) (**W**^{2250}CHDNGVNYKIGEKWDR), C) Spectrum of the di-Trp cross-linked peptide (**W**^{445}CGTTQNYDADQK) (**W**^{2264}DRQGENGQMMSCTCLGNGK). Spectra were obtained from the software MassAI. Peaks matching y and z fragment ions are indicated in blue, whereas those matching blue b and c ions are shown in green. Peaks in red are unassigned ions.

```
        10         20         30         40         50         60         70         80         90        100
MLRGPGPGLL LLAVQCLGTA VPSTGASKSK RQAQQMVQPQ SPVAVSQSKP GCYDNGKHYQ INQQWERTYL GNALVCTCYG GSRGFNCESK PEAEETCFDK
       110        120        130        140        150        160        170        180        190        200
```

YTGNTYRVGD TYERPKDSMI WDCTCIGAGR GRISCTIANR CHEGGQSYKI GDTWRRPHET GGYMLECVCL GNGKGEWTCK PIAEKCFDHA AGTSYVVGET

210 220 230 240 250 260 270 280 290 300

WEKPYQGWMM VDCTCLGEGS GRITCTSRNR CNDQDTRTSY RIGDTWSKKD NRGNLLQCIC TGNGRGEWKC ERHTSVQTTS SGSGPFTDVR AAVYQPQPHP

310 320 330 340 350 360 370 380 390 400

QPPPYGHCVT DSGVVYSVGM QWLKTQGNKQ MLCTCLGNGV SCQETAVTQT YGGNSNGEPC VLPFTYNGRT FYSCTTEGRQ DGHLWCSTTS NYEQDQKYSF

410 420 430 440 450 460 470 480 490 500

CTDHTVLVQT RGGNSNGALC HFPFLYNNHN YTDCTSEGRR DNMKWCGTTQ NYDADQKFGF CPMAAHEEIC TTNEGVMYRI GDQWDKQHDM GHMMRCTCVG

510 520 530 540 550 560 570 580 590 600

NGRGEWTCIA YSQLRDQCIV DDITYNVNDT FHKRHEEGHM LNCTCFGQGR GRWKCDPVDQ CQDSETGTFY QIGDSWEKYV HGVRYQCYCY GRGIGEWHCQ

610 620 630 640 650 660 670 680 690 700

PLQTYPSSSG PVEVFITETP SQPNSHPIQW NAPQPSHISK YILRWRPKNS VGRWKEATIP GHLNSYTIKG LKPGVVYEGQ LISIQQYGHQ EVTRFDFTTT

710 720 730 740 750 760 770 780 790 800

STSTPVTSNT VTGETTPFSP LVATSESVTE ITASSFVVSW VSASDTVSGF RVEYELSEEG DEPQYLDLPS TATSVNIPDL LPGRKYIVNV YQISEDGEQS

810 820 830 840 850 860 870 880 890 900

LILSTSQTTA PDAPPDTTVD QVDDTSIVVR WSRPQAPITG YRIVYSPSVE GSSTELNLPE TANSVTLSDL QPGVQYNITI YAVEENQEST PVVIQQETTG

910 920 930 940 950 960 970 980 990 1000

TPRSDTVPSP RDLQFVEVTD VKVTIMWTPP ESAVTGYRVD VIPVNLPGEH GQRLPISRNT FAEVTGLSPG VTYYFKVFAV SHGRESKPLT AQQTTKLDAP

1010 1020 1030 1040 1050 1060 1070 1080 1090 1100

TNLQFVNETD STVLVRWTPP RAQITGYRLT VGLTRRGQPR QYNVGPSVSK YPLRNLQPAS EYTVSLVAIK GNQESPKATG VFTTLQPGSS IPPYNTEVTE

1110 1120 1130 1140 1150 1160 1170 1180 1190 1200

TTIVITWTPA PRIGFKLGVR PSQGGEAPRE VTSDSGSIVV SGLTPGVEYV YTIQVLRDGQ ERDAPIVNKV VTPLSPPTNL HLEANPDTGV LTVSWERSTT

1210 1220 1230 1240 1250 1260 1270 1280 1290 1300

PDITGYRITT TPTNGQQGNS LEEVVHADQS SCTFDNLSPG LEYNVSVYTV KDDKESVPIS DTIIPAVPPP TDLRFTNIGP DTMRVTWAPP PSIDLTNFLV

1310 1320 1330 1340 1350 1360 1370 1380 1390 1400

RYSPVKNEED VAELSISPSD NAVVLTNLLP GTEYVVSVSS VYEQHESTPL RGRQKTGLDS PTGIDFSDIT ANSFTVHWIA PRATITGYRI RHHPEHFSGR

1410 1420 1430 1440 1450 1460 1470 1480 1490 1500

PREDRVPHSR NSITLTNLTP GTEYVVSIVA LNGREESPLL IGQQSTVSDV PRDLEVVAAT PTSLLISWDA PAVTVRYYRI TYGETGGNSP VQEFTVPGSK

1510 1520 1530 1540 1550 1560 1570 1580 1590 1600

STATISGLKP GVDYTITVYA VTGRGDSPAS SKPISINYRT EIDKPSQMQV TDVQDNSISV KWLPSSSPVT GYRVTTTPKN GPGPTKTKTA GPDQTEMTIE

1610 1620 1630 1640 1650 1660 1670 1680 1690 1700

GLQPTVEYVV SVYAQNPSGE SQPLVQTAVT NIDRPKGLAF TDVDVDSIKI AWESPQGQVS RYRVTYSSPE DGIHELFPAP DGEEDTAELQ GLRPGSEYTV

1710 1720 1730 1740 1750 1760 1770 1780 1790 1800

SVVALHDDME SQPLIGTQST AIPAPTDLKF TQVTPTSLSA QWTPPNVQLT GYRVRVTPKE KTGPMKEINL APDSSSVVVS GLMVATKYEV SVYALKDTLT

1810 1820 1830 1840 1850 1860 1870 1880 1890 1900

SRPAQGVVTT LENVSPPRRA RVTDATETTI TISWRTKTET ITGFQVDAVP ANGQTPIQRT IKPDVRSYTI TGLQPGTDYK IYLYTLNDNA RSSPVVIDAS

1910 1920 1930 1940 1950 1960 1970 1980 1990 2000

TAIDAPSNLR FLATTPNSLL VSWQPPRARI TGYIIKYEKP GSPPREVVPR PRPGVTEATI TGLEPGTEYT IYVIALKNNQ KSEPLIGRKK TDELPQLVTL

2010 2020 2030 2040 2050 2060 2070 2080 2090 2100

PHPNLHGPEI LDVPSTVQKT PFVTHPGYDT GNGIQLPGTS GQQPSVGQQM IFEEHGFRRT TPPTTATPIR HRPRPYPPNV GEEIQIGHIP REDVDYHLYP

2110 2120 2130 2140 2150 2160 2170 2180 2190 2200

HGPGLNPNAS TGQEALSQTT ISWAPFQDTS EYIISCHPVG TDEEPLQFRV PGTSTSATLT GLTRGATYNV IVEALKDQQR HKVREEVVTV GNSVNEGLNQ

2210 2220 2230 2240 2250 2260 2270 2280 2290 2300

```
PTDDSCFDPY TVSHYAVGDE WERMSESGFK LLCQCLGFGS GHFRCDSSRW  CHDNGVNYKI GEKWDRQGEN GQMMSCTCLG NGKGEFKCDP HEATCYDDGK

      2310       2320       2330       2340       2350        2360       2370       2380

TYHVGEQWQK EYLGAICSCT CFGGQRGWRC DNCRRPGGEP SPEGTTGQSY  NQYSQRYHQR TNTNVNCPIE CFMPLDVQAD REDSRE
```

Supplementary Figure 4. Primary sequence of isoform 1 of human plasma fibronectin. Modified residues are highlighted in bold, and color coded corresponding to the extent of modification for the 1 μM FN, 50 μM ONOOH and 150 mM NaCl condition. Residues involved in cross-links are underlined. Y and W residues highlighted in red correspond to level of nitration higher than 30%; orange corresponds to a level of nitration of 20 - 30%; yellow corresponds to 10 - 20%; and blue corresponds to a level of 1 - 10% nitration. Green coloration corresponds to no detectable nitration; purple coloration corresponds to a residue that was not detected. M residues in red were detected as the oxidized (+16, sulfoxide) species.

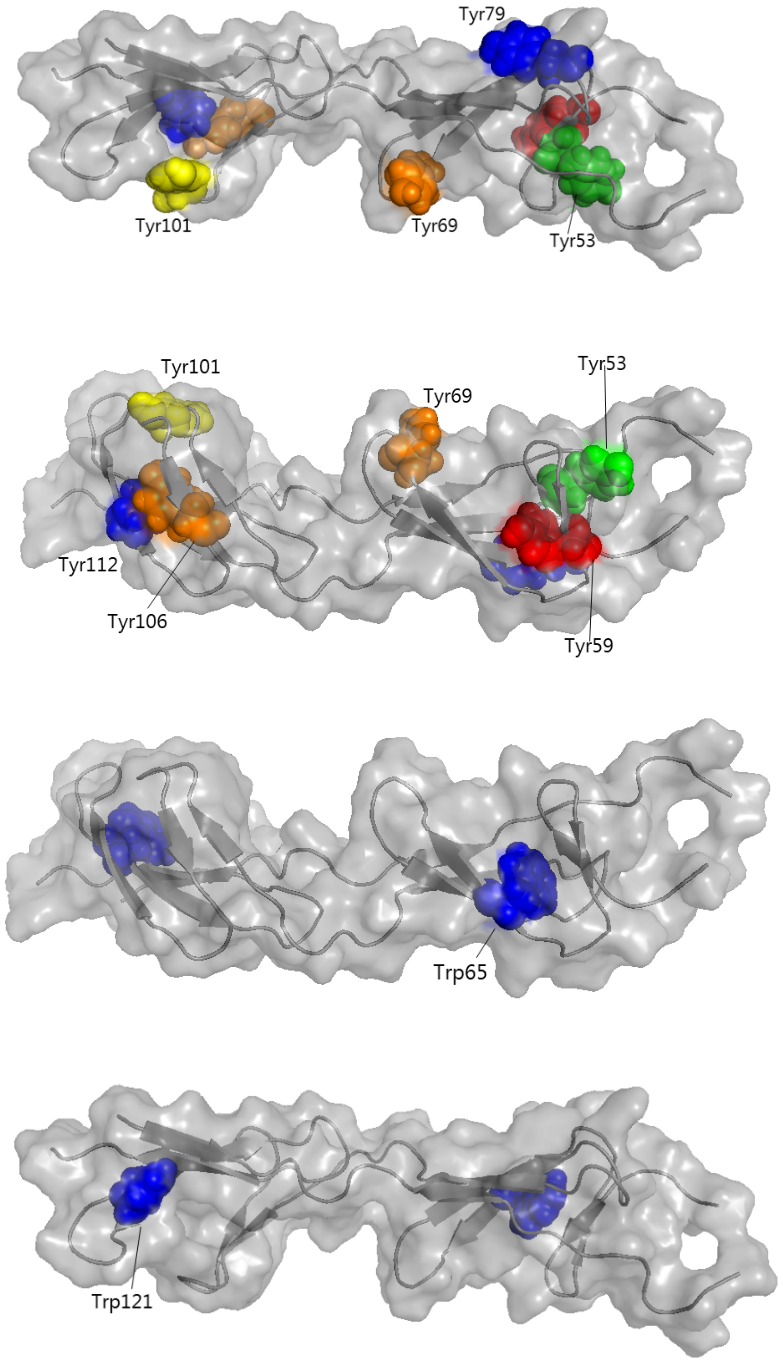

Supplementary Figure 5. Rendering of Tyr and Trp residues identified as nitrated in FN treated with 50 μM ONOOH (150 mM NaCl). Red color corresponds to level of nitration higher than 30%; orange to nitration between 20 - 30%; yellow to nitration between 10 - 20%; and blue color corresponds to between 1 - 10% nitration. Green color corresponds to no detectable nitration. PDB structure: 1o9a (65), corresponding to domains $FNI_{1\text{-}3}$ and residues 48-140.

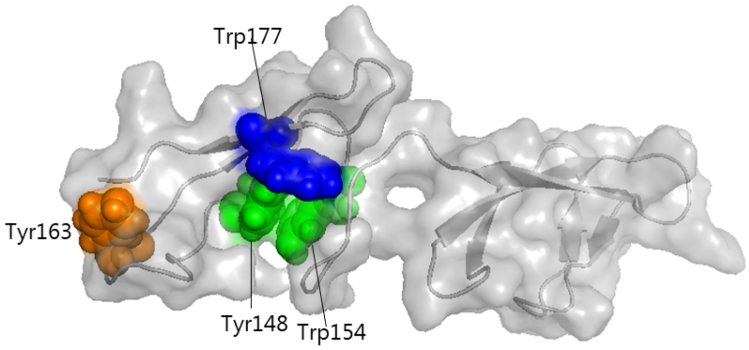

Supplementary Figure 6. Rendering of Tyr and Trp residues identified as nitrated in FN treated with 50 μM ONOOH (150 mM NaCl). Red color corresponds to level of nitration higher than 30%; orange to nitration between 20 - 30%; yellow to nitration between 10 - 20%; and blue color corresponds to between 1 - 10% nitration. Green color corresponds to no detectable nitration. PDB structure: 2cku (63), corresponding to domains FNI_{2-3} and residues 93-182.

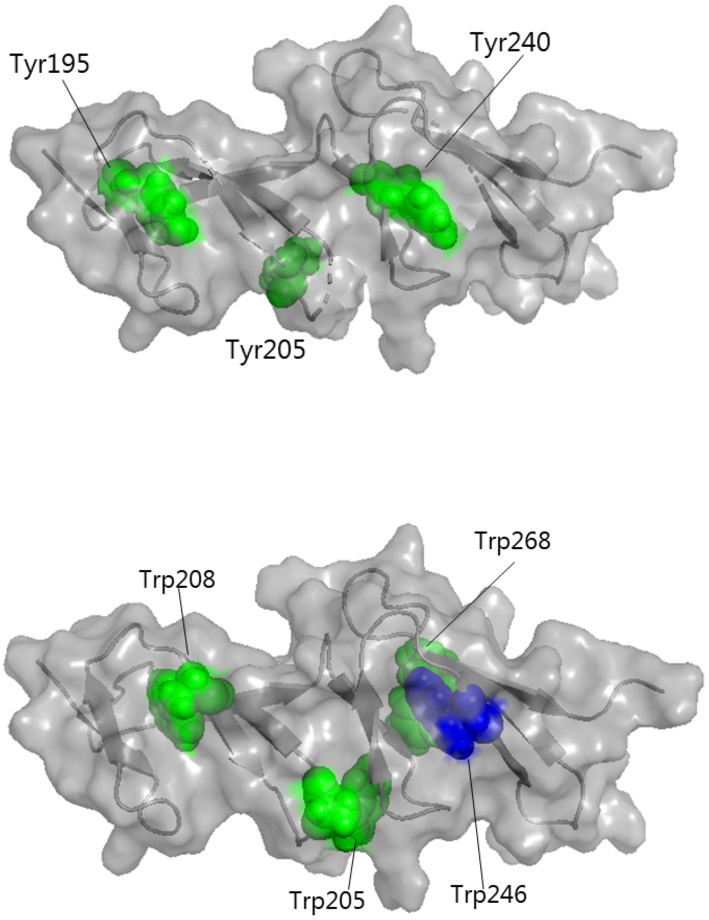

Supplementary Figure 7. Rendering of Tyr and Trp residues identified as nitrated in FN treated with 50μM ONOOH (150 mM NaCl). Red color corresponds to level of nitration higher than 30%; orange to nitration between 20 - 30%; yellow to nitration between 10 - 20%; and blue color corresponds to between 1 - 10% nitration. Green color corresponds to no detectable nitration. PDB structure: 1fbr (66) corresponding to domains FNI_{4-5} and residues 183-275.

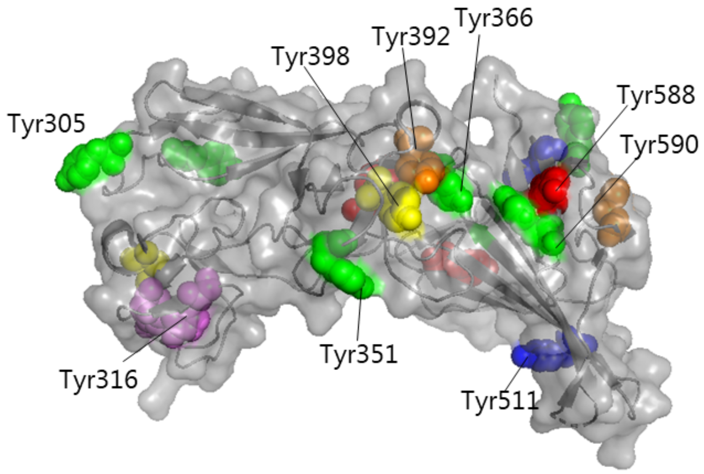
Tyr305
Tyr398
Tyr392
Tyr366
Tyr588
Tyr590
Tyr316
Tyr351
Tyr511

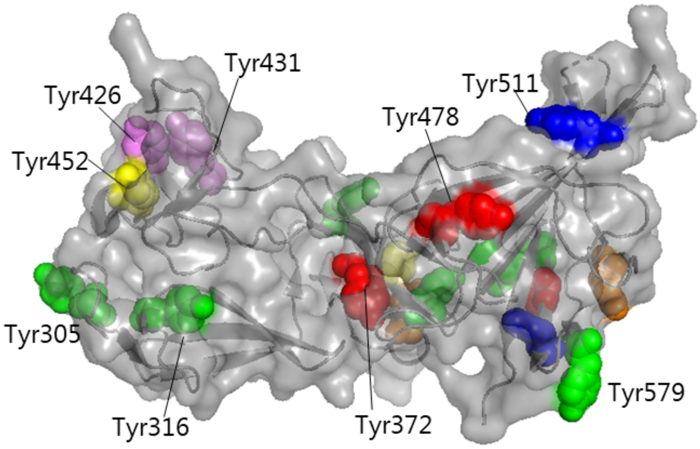
Tyr431
Tyr426
Tyr452
Tyr511
Tyr478
Tyr305
Tyr316
Tyr372
Tyr579

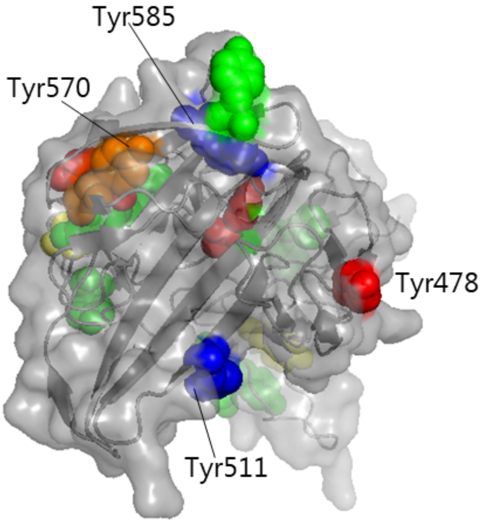
Tyr585
Tyr570
Tyr478
Tyr511

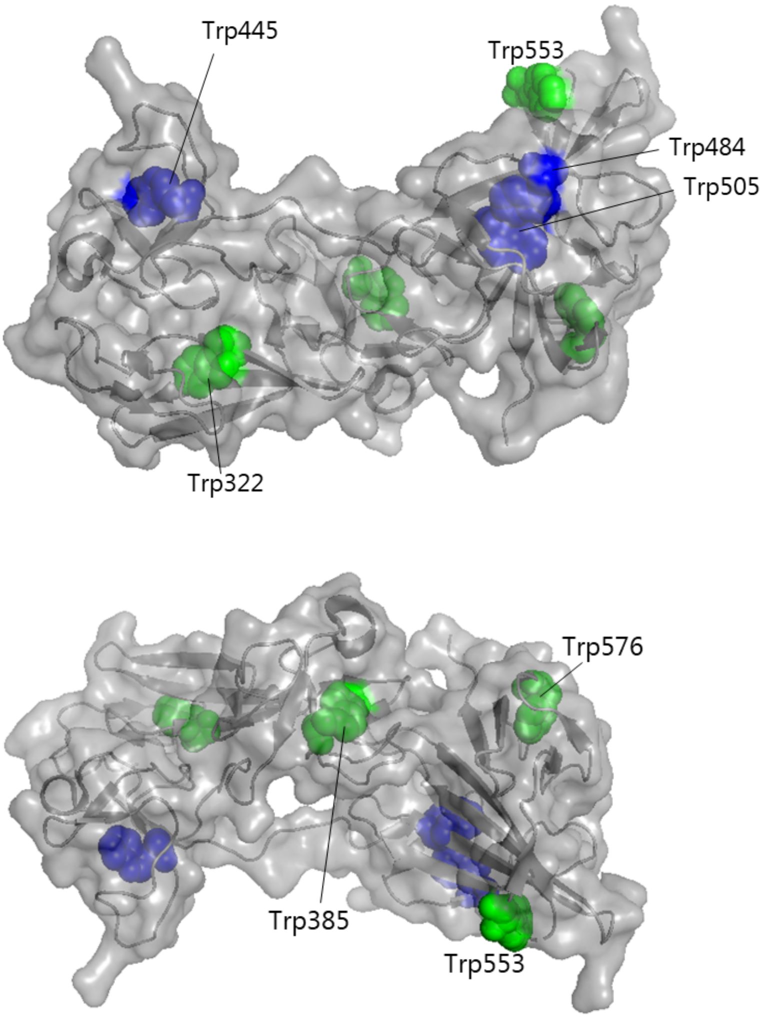

Supplementary Figure 8. Rendering of Tyr and Trp residues identified as nitrated in FN treated with 50μM ONOOH (150 mM NaCl). Red color corresponds to level of nitration higher than 30%; orange to nitration between 20 - 30%; yellow to nitration between 10 - 20%; and blue color corresponds to between 1 - 10% nitration. Green color corresponds to no detectable nitration. The purple color indicates that the residue was not detected in the tryptic digests. PDB structure: 3m7p (56) corresponding to domains FNI_6, $FNII_{1-2}$, FNI_{7-9} and residues 297-604.

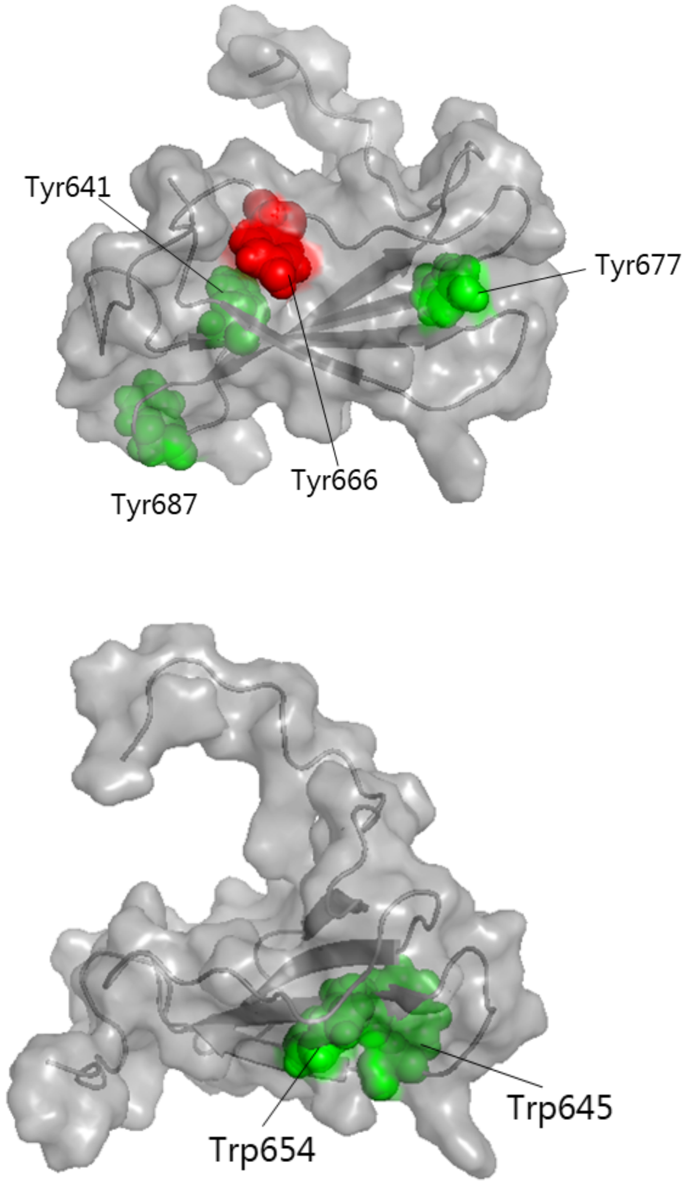

Supplementary Figure 9. Rendering of Tyr and Trp residues identified as nitrated in FN treated with 50μM ONOOH (150 mM NaCl). Red color corresponds to level of nitration higher than 30%; orange to nitration between 20 - 30%; yellow to nitration between 10 - 20%; and blue color corresponds to between 1 - 10% nitration. Green color corresponds to no detectable nitration. PDB structure: 1q38 (67), corresponding to $FNIII_1$ and residues 631-705.

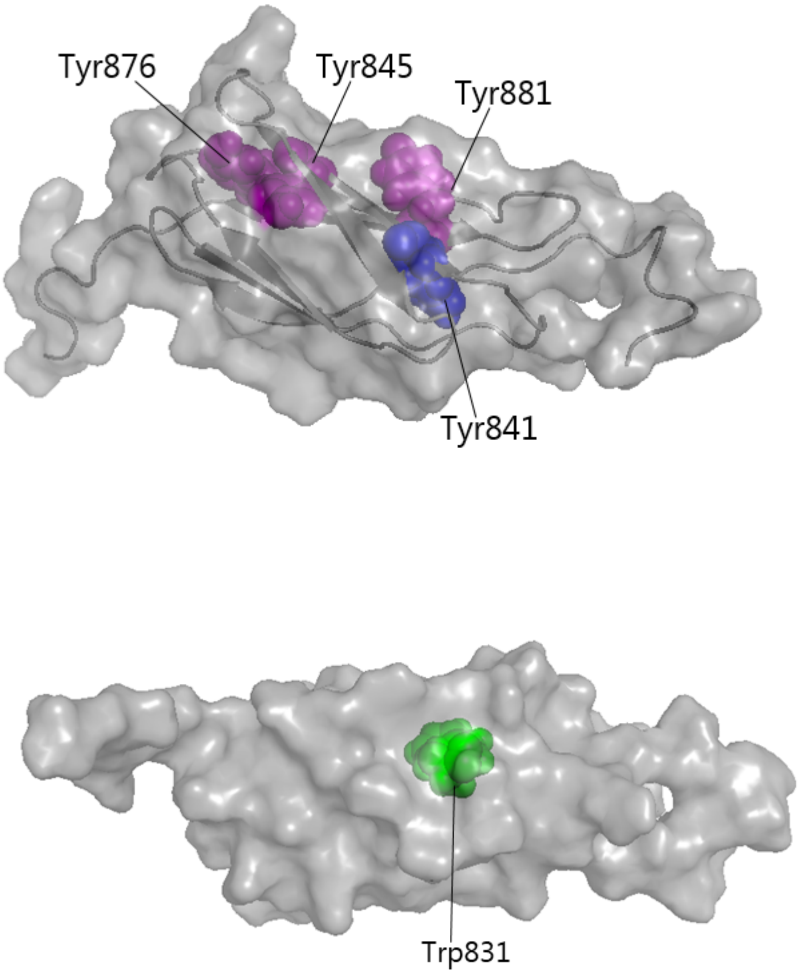

Supplementary Figure 10. Rendering of Tyr and Trp residues identified as nitrated in FN treated with 50μM ONOOH (150 mM NaCl). Red color corresponds to level of nitration higher than 30%; orange to nitration between 20 - 30%; yellow to nitration between 10 - 20%; and blue color corresponds to between 1 - 10% nitration. Green color corresponds to no detectable nitration. The purple color indicates that the residue was not detected in the tryptic digests. PDB structure: 2N1K (68) corresponding to domains $FNIII_{2,3}$ and residues 808-905.

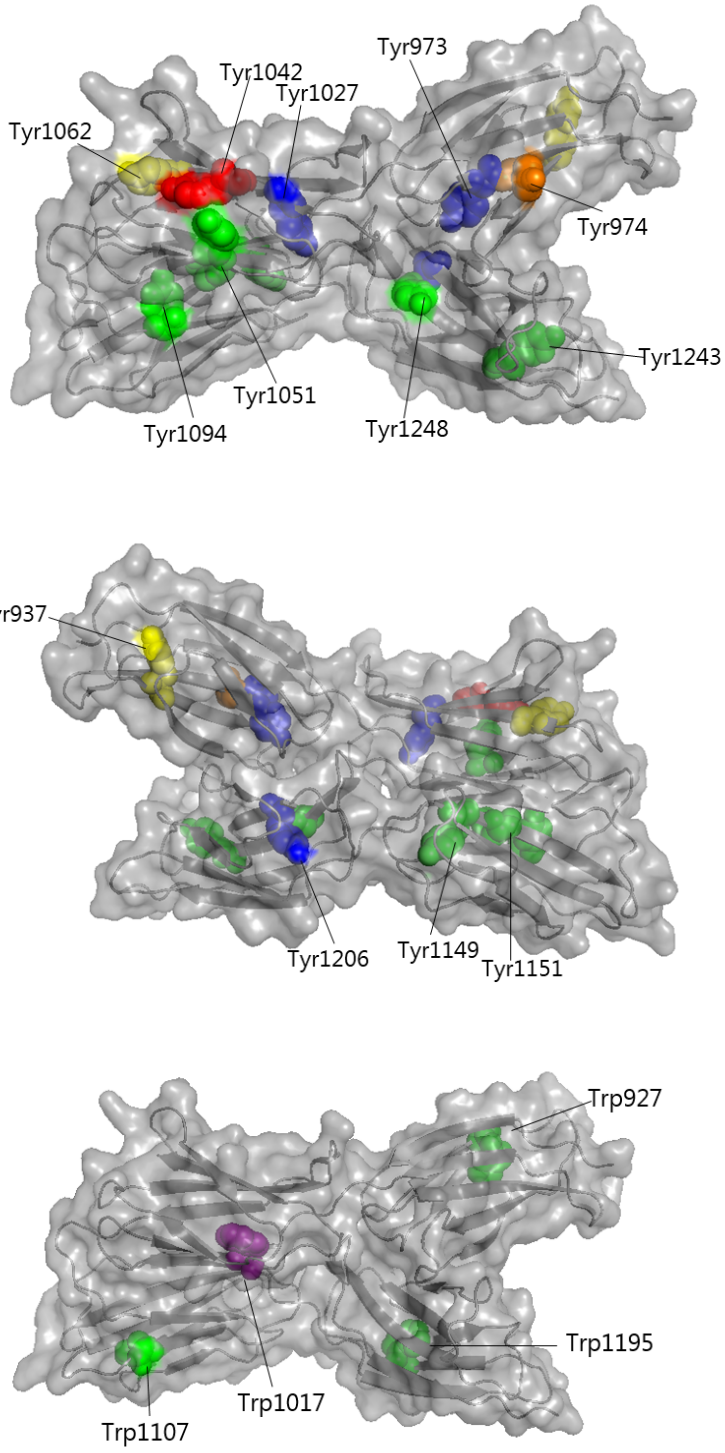

Supplementary Figure 11. Rendering of Tyr and Trp residues identified as nitrated in FN treated with 50μM ONOOH (150 mM NaCl). Red color corresponds to level of nitration higher than 30%; orange to nitration between 20 - 30%; yellow to nitration between 10 - 20%; and blue color corresponds to between 1 - 10% nitration. Green color corresponds to no detectable nitration. The purple color indicates that the residue was not detected in the tryptic digests. PDB structure: 6mfa (unpublished data deposited in PDB), corresponding to domains $FNIII_{3-7}$, and residues 903-1268.

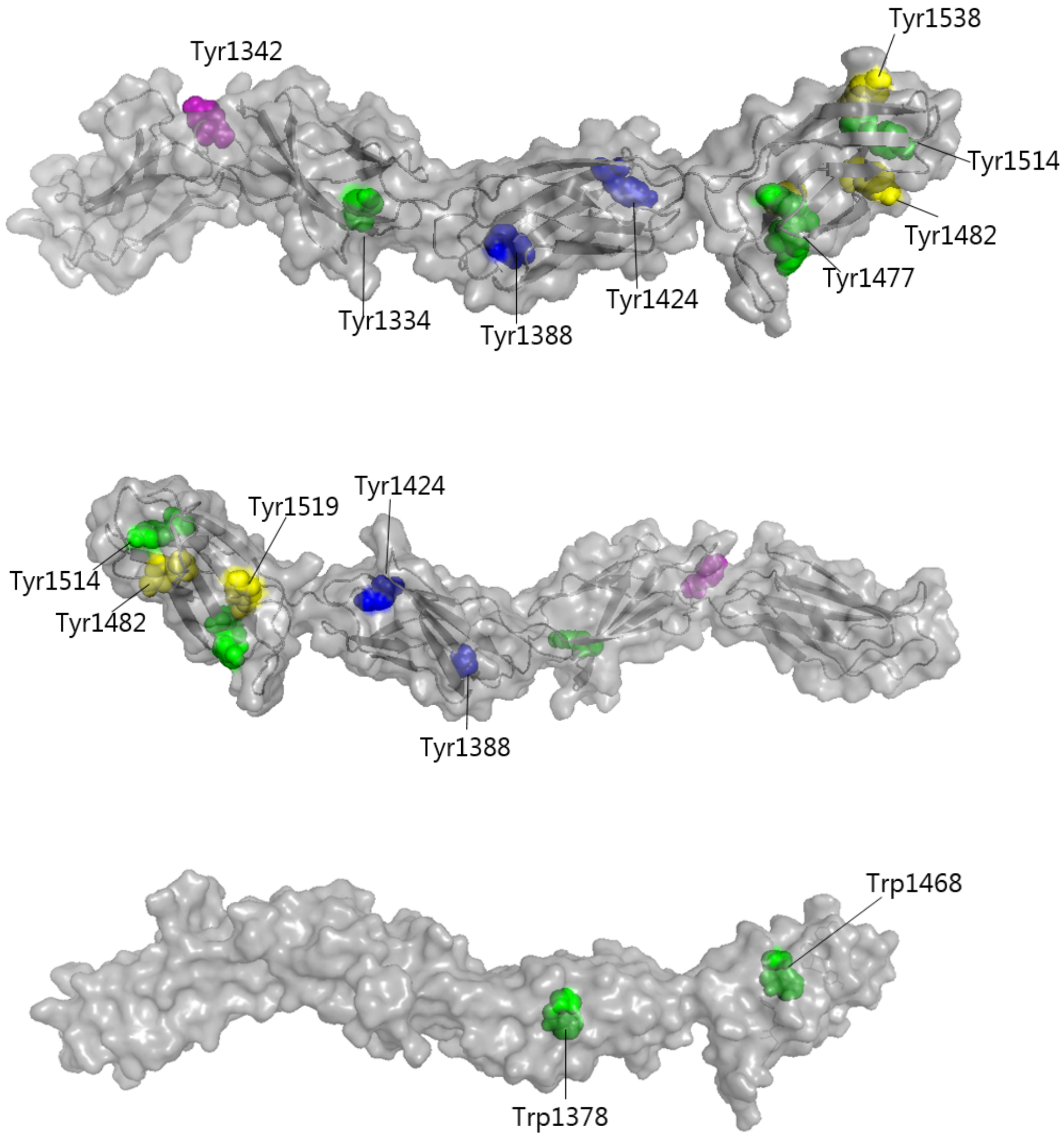

Supplementary Figure 12. Rendering of Tyr and Trp residues identified as nitrated in FN treated with 50μM ONOOH (150 mM NaCl). Red color corresponds to level of nitration higher than 30%; orange to nitration between 20 - 30%; yellow to nitration between 10 - 20%; and blue color corresponds to between 1 - 10% nitration. Green color corresponds to no detectable nitration. The purple color indicates that the residue was not detected in the tryptic digests. PDB structure: 1fnf (69) corresponding to domains $FNIII_{6-10}$ and residues 1173-1540.

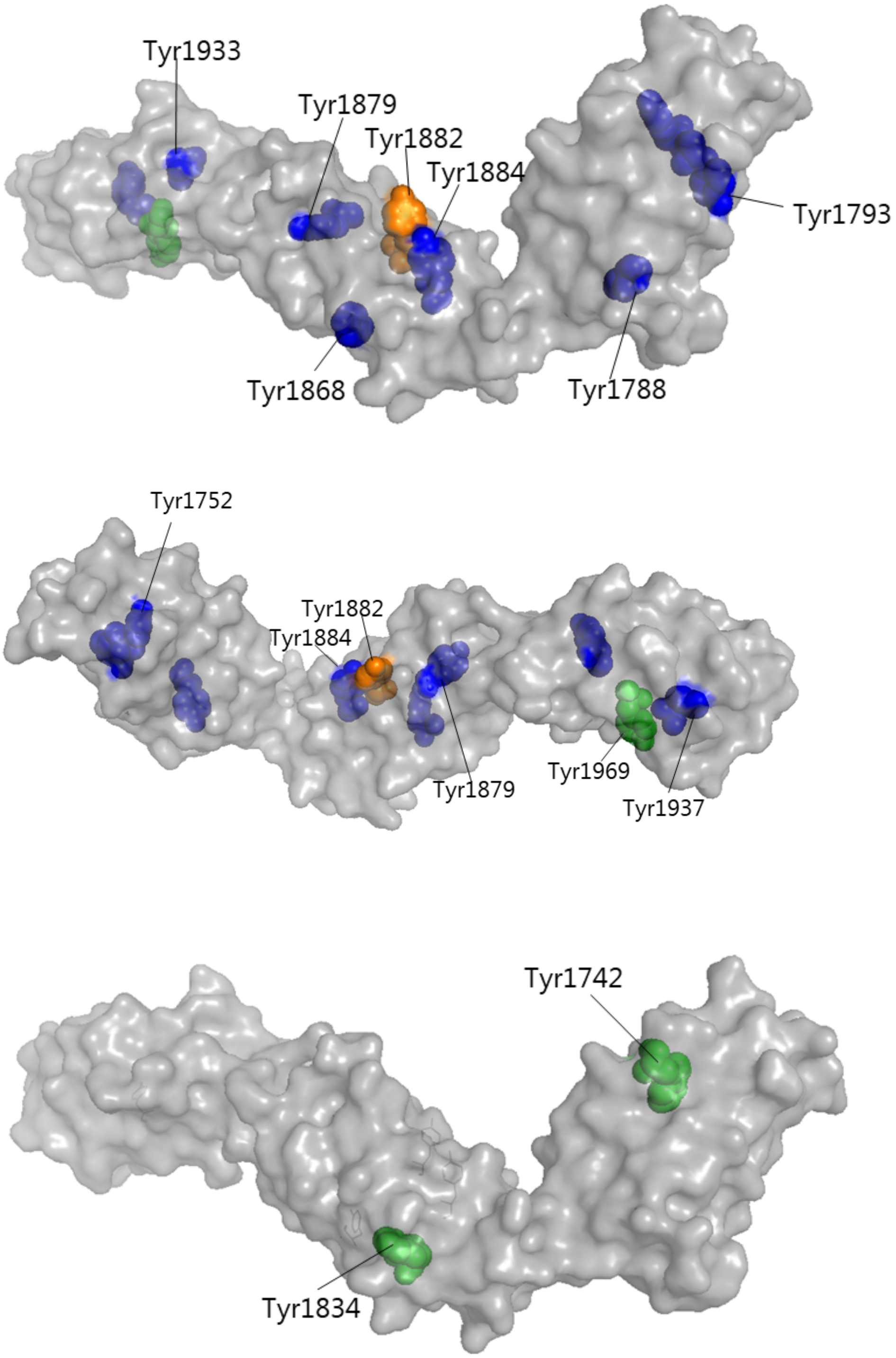

Supplementary Figure 13. Rendering of Tyr and Trp residues identified as nitrated in FN treated with 50μM ONOOH (150 mM NaCl). Red color corresponds to level of nitration higher than 30%; orange to nitration between 20 - 30%; yellow to nitration between 10 - 20%; and blue color corresponds to between 1 - 10% nitration. Green color corresponds to no detectable nitration. PDB structure: 3r8q (unpublished data deposited in PDB) corresponding to domains $FNIII_{12-15}$, and residues 1721-1991.

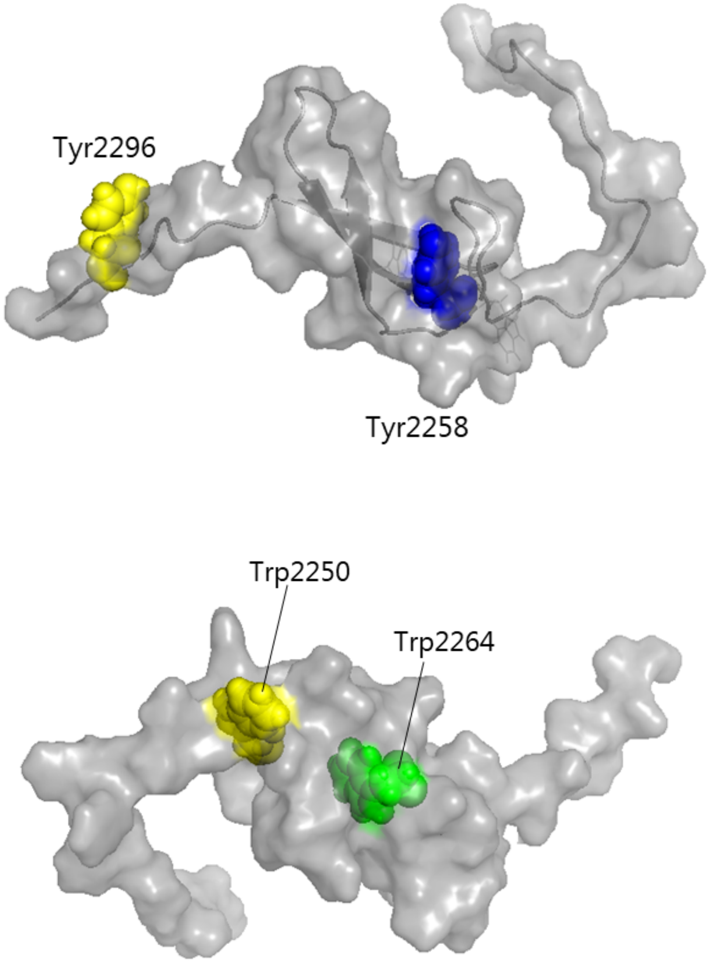

Supplementary Figure 14. Rendering of Tyr and Trp residues identified as nitrated in FN treated with 50μM ONOOH (150 mM NaCl). Red color corresponds to level of nitration higher than 30%; orange to nitration between 20 - 30%; yellow to nitration between 10 - 20%; and blue color corresponds to between 1 - 10% nitration. Green color corresponds to no detectable nitration, although Trp2264 was detected as nitrated in a cross-linked peptide, see Table 2). PDB structure: 2ec3 (unpublished data deposited in PDB) corresponding to domain FNI_{11} (residues 2241-2299).
